# Systems Level Analysis of Systemic Sclerosis Shows a Network of Immune and Profibrotic Pathways Connected with Genetic Polymorphisms

**DOI:** 10.1371/journal.pcbi.1004005

**Published:** 2015-01-08

**Authors:** J. Matthew Mahoney, Jaclyn Taroni, Viktor Martyanov, Tammara A. Wood, Casey S. Greene, Patricia A. Pioli, Monique E. Hinchcliff, Michael L. Whitfield

**Affiliations:** 1 Department of Genetics, Geisel School of Medicine at Dartmouth, Hannover, New Hampshire, United States of America; 2 Department of Obstetrics and Gynecology, Geisel School of Medicine at Dartmouth, Hannover, New Hampshire, United States of America; 3 Department of Medicine, Northwestern University Feinberg School of Medicine, Chicago, Illinois, United States of America; University of Texas Health Science Center at Houston, United States of America

## Abstract

Systemic sclerosis (SSc) is a rare systemic autoimmune disease characterized by skin and organ fibrosis. The pathogenesis of SSc and its progression are poorly understood. The SSc intrinsic gene expression subsets (inflammatory, fibroproliferative, normal-like, and limited) are observed in multiple clinical cohorts of patients with SSc. Analysis of longitudinal skin biopsies suggests that a patient's subset assignment is stable over 6–12 months. Genetically, SSc is multi-factorial with many genetic risk loci for SSc generally and for specific clinical manifestations. Here we identify the genes consistently associated with the intrinsic subsets across three independent cohorts, show the relationship between these genes using a gene-gene interaction network, and place the genetic risk loci in the context of the intrinsic subsets. To identify gene expression modules common to three independent datasets from three different clinical centers, we developed a consensus clustering procedure based on mutual information of partitions, an information theory concept, and performed a meta-analysis of these genome-wide gene expression datasets. We created a gene-gene interaction network of the conserved molecular features across the intrinsic subsets and analyzed their connections with SSc-associated genetic polymorphisms. The network is composed of distinct, but interconnected, components related to interferon activation, M2 macrophages, adaptive immunity, extracellular matrix remodeling, and cell proliferation. The network shows extensive connections between the inflammatory- and fibroproliferative-specific genes. The network also shows connections between these subset-specific genes and 30 SSc-associated polymorphic genes including *STAT4*, *BLK*, *IRF7*, *NOTCH4*, *PLAUR*, *CSK*, *IRAK1*, and several human leukocyte antigen (HLA) genes. Our analyses suggest that the gene expression changes underlying the SSc subsets may be long-lived, but mechanistically interconnected and related to a patients underlying genetic risk.

## Author Summary

Systemic sclerosis (SSc) is a rare autoimmune disease characterized by skin thickening (fibrosis) and progressive organ failure. Previous studies of SSc skin biopsies have identified molecular subsets of SSc based upon gene expression termed the inflammatory, fibroproliferative, normal-like, and limited intrinsic subsets. These gene expression signatures are large and although the biological processes are conserved, the exact list of genes can vary across datasets due to random variation, as well as minor differences in the composition of the study cohorts (e.g. early vs. late disease). We developed a computational tool to identify the consensus genes underlying the subsets across heterogeneous data and characterized the biological role of the consensus genes in SSc in order to obtain a systems level perspective of the SSc subsets. Our analysis reveals a complex network of genes connecting two of the major SSc intrinsic subsets, inflammatory and fibroproliferative. Many genetic loci associated with SSc risk show connections with the consensus genes of the intrinsic subsets, indicating that differential expression of genes defining the subsets may be related to genetic risk for SSc, thus for the first time placing the genetic risk factors in the context of, and showing putative relationships with, the intrinsic gene expression subsets.

## Introduction

Genome-scale gene expression profiling of systemic sclerosis (SSc) skin has identified distinct intrinsic molecular subsets (inflammatory, fibroproliferative, and normal-like) within the subset of patients diagnosed with diffuse cutaneous SSc (dSSc) based upon the extent of skin involvement. These subsets are identified by an intrinsic gene analysis [1] that shifts the focus to differences between patients rather than patient biopsies. The inflammatory subset is characterized by increased expression of genes associated with inflammation and extracellular matrix (ECM) deposition, while the fibroproliferative subset is characterized by increased expression of genes associated with cell proliferation [1], [2]. Biopsies from patients in the normal-like subset show gene expression most similar to healthy control skin biopsies. The presence of three distinct molecular SSc subsets within patients diagnosed with dSSc underscores the molecular heterogeneity of SSc. However, it is unclear whether the subsets represent distinct diseases with different etiologies or whether they represent disease progression. To address this question, we identified the conserved molecular pathways characteristic of each subset that are reproducible between different datasets from multiple patient cohorts, and examined the connectivity of these genes and SSc-associated polymorphic genes in a predicted functional network.

SSc is a rare disease without validated disease progression markers and no known cure. SSc affects between 49,000–276,000 Americans; one in three patients dies within 10 years of diagnosis [3]. The rarity of SSc makes this disease an excellent test case for a genomic meta-analysis to understand disease mechanism. Using this approach, we have begun to understand the molecular and clinical complexity of SSc. Our findings may assist in generating patient-specific therapies [4] and delivering real-time quantitative feedback regarding therapeutic response during clinical trials [4], [5].

High-throughput gene expression data has demonstrated that genes that function together are almost always co-expressed and thus highly correlated with each other [6], [7]. Indeed, the expressed genes in a biopsy form a co-expression network, where genes serve as nodes and correlations as links between genes. (The language of networks is technical and beyond the scope of this paper. The supporting information (S1 Text) contains a glossary of keywords that are used in a technical sense in the main body of the paper.) This observation forms part of the basis for “network medicine” [8], [9]. The co-expression network contains groups of highly correlated genes that represent the biological processes at work in the tissue [6], [10]. These groups of genes can be found using a variety of procedures for co-expression clustering (e.g. [6], [10]). All of these procedures group highly correlated genes together, i.e. they partition the genome into non-overlapping groups of genes with similar expression patterns sometimes called modules. The output of co-expression clustering is a data-driven partition of the expressed genes in the genome. As a result of technical differences in data acquisition protocols as well as true biological variation (patient heterogeneity, treatment), the exact modules identified in one SSc dataset differ somewhat from those identified in another dataset, although the same pathways, biological processes, and many core genes are found in each dataset. We developed a tool to compare gene co-expression modules derived from multiple disparate datasets to identify the modules that are reproducibly expressed in each dataset. We then derived gene sets from the overlaps between conserved modules across datasets. These “consensus clusters” are the gene clusters that are conserved across all datasets.

A naive approach to solving this problem is to simply intersect the “intrinsic gene lists” derived for each of the cohorts [1], [4], [11]. The methodological issue with this approach is that these lists are derived under a large multiple hypothesis testing burden, and although the same biological processes and some genes are found reproducibly, the gene sets do not exactly recapitulate across data sets [11]. This simple intersection approach would be much too conservative and consequently exclude many biologically important genes. Our alternative approach is to consider “modules first”. In brief, our goal is to identify the modules that are conserved across datasets first and then extract the consensus genes as those that are consistently assigned to those modules. This transfers the multiple testing burden onto the much smaller list of modules and allows genes to be included in the consensus even if they do not achieve extremely high statistical significance in all datasets simultaneously.

We developed this idea into a novel data mining procedure called Mutual Information Consensus Clustering (MICC) to identify conserved gene expression modules across multiple gene expression datasets. Consensus clustering is a set of techniques from computer science and bioinformatics that refers to strategies for extracting robust clusters from an *ensemble* of partitions. Typically this is done using a large ensemble of partitions. Early work focused on weak clustering algorithms and consensus clustering was used to “boost” the weak partitions into an aggregate, consensus partition [12]. In bioinformatics, consensus clustering algorithms have been developed to aggregate ensembles of partitions that are derived from data resampling [13]. These techniques have in common that they do not “trust” a particular partition from one of their clustering algorithms. Here, we use a strong clustering algorithm called weighted gene co-expression network analysis (WGCNA). Our ensemble of partitions is the collection that we obtain from having multiple, clustered datasets from independent cohorts. While WGCNA extracts meaningful signals in each data set, the potentially interesting modules in one dataset are not precisely replicated in all others.

Mutual information [14] provides a rigorous criterion by which modules from different datasets can be said to have significant overlap (i.e. are conserved) and allows one to identify when the available information between two partitions is exhausted. Mutual information is a sum of positive and negative contributions from each pair of modules across datasets, and MICC automatically disregards all overlaps that do not contribute positively to the total mutual information, thus giving an objective measure of conserved gene expression that is both comprehensive and parsimonious. As such, MICC is a metaclustering procedure that “clusters the clusters” [12], but does not produce a complete partition. Instead, it generates only a partition of the subset of the genome that has strongly conserved gene co-expression.

Using previously published gene expression data from skin biopsies from patients with SSc recruited at three independent academic centers [1], [4], [11] and new samples analyzed as part of this study (Table 1), we identified the consensus clusters that were present in all datasets. Due to the unbiased nature of high-throughput screening, these datasets contain information about SSc-specific biology as well as the general biology of skin. We showed that MICC yields consensus clusters that are biologically specific. At the level of the whole transcriptome, we demonstrated that the consensus clusters are enriched for hubs defined by co-expression network analysis. We then filtered the consensus clusters down to those that were intrinsic subset-specific. The existence of the intrinsic subsets is a robust observation in each of these studies and the consensus clusters associated with them provide a rigorous picture of the core gene set underlying the subsets. The comprehensive and concise annotation of the conserved differential gene expression that we developed suggests that the intrinsic subsets represent pathophysiological states of one disease. Our major findings include the following: 1. We show that the subset-specific consensus clusters are part of a gene-gene network and for the first time to our knowledge, demonstrate putative connections between the intrinsic gene expression subsets of SSc and SSc-associated genetic polymorphisms identified by candidate and genome-wide association studies (GWAS). 2. We provide additional unbiased data to support the hypothesis that immune system activation is an early event and plays a central role in SSc pathogenesis as SSc risk alleles are linked to the immune system nodes of our network. 3. The consensus gene-gene network provides insights into genes that may be central to the major disease processes and identifies genes and pathways that may connect these major groups of genes. 4. We show a link between the inflammatory and fibroproliferative patient groups through a shared TGFβ/ECM subnetwork, suggesting a theoretical path by which these gene expression subsets may be linked. Collectively, these findings demonstrate that MICC is a powerful tool that identifies the reproducible signals in gene expression data across multiple datasets and shows how they may relate to the genetic polymorphisms associated with SSc.

**Table 1:** Composition of study cohorts.

Data Set	Pendergrass		Milano			Hinchcliff		
	SSc	Control	SSc	Control	Morphea	SSc	Control	Morphea
All subjects	N = 22	N = 9	N = 24	N = 6	N = 3	N = 34	N = 11	N = 1
Age*, mean (SD)	46.1 (9.1)	NA	51.8 (10.4)	40.2 (10.6)	50.7 (2.9)	47.7 (11.7)	41.2 (11.8)	47
Sex, n (% women)	17 (77.3)	NA	21 (87.5)	5 (83.3)	3 (100.0)	32 (94.1)	7 (63.6)	1 (100.0)
SSc patients only	N = 22		N = 24			N = 34		
SSc subtype, n (% diffuse)	22 (100.0)		17 (70.8)			31 (91.2)		
SSc disease duration*, mean (SD)	18.4 (13.1) mos		7.7 (7.2) yrs			52.7 (67.4) mos		
SSc autoantibody, n (% positive)								
Scl-70	3 (13.6)		5 (20.8)			9 (26.5)		
RNA Pol III	3 (13.6)		NA			9 (26.5)		
ACA	1 (4.5)		2 (8.3)			2 (5.9)		
SSc intrinsic subset, n (%)								
Inflammatory	9 (40.9)		5 (20.8)			17 (50.0)		
Proliferative	8 (36.4)		11 (45.8)			7 (20.6)		
Normal-like	2 (9.1)		4 (16.7)			7 (20.6)		
Limited	NA		3 (12.5)			2 (5.9)		

*At base biopsy.

The three cohorts of patients with SSc analyzed in this study: Milano et al. [1], Pendergrass et al. [11], and an expanded version of Hinchcliff et al. [4].

## Results

**Figure 1:**
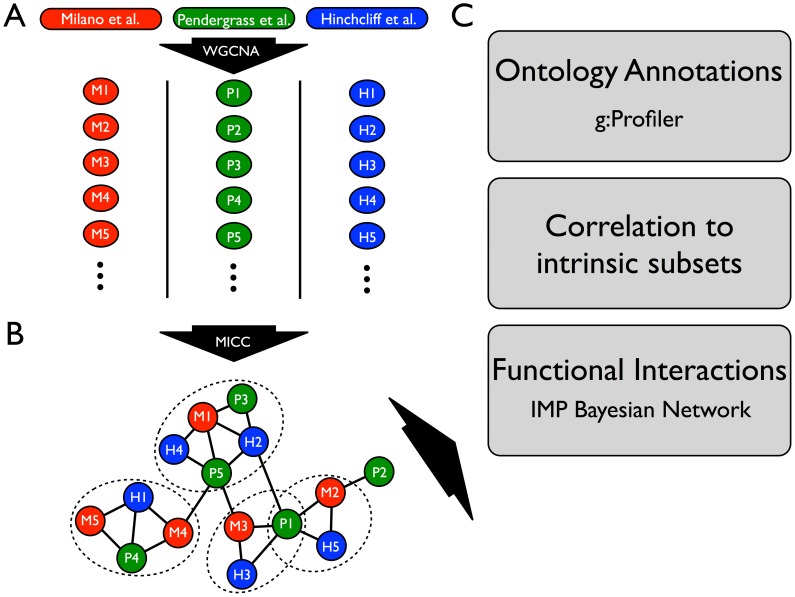
Schematic of the analysis pipeline for integrative analysis of multiple SSc skin datasets. (**A**) Each microarray dataset (Milano et al., Pendergrass et al., and Hinchcliff et al.) was independently clustered by WGCNA into gene coexpression modules (colored circles). Each module is a set of genes that was highly correlated within a dataset. (**B**) Modules were compared across datasets using a novel procedure (MICC) to determine which were approximately conserved across all three datasets. The network in (**B**) is called the *information graph* and encodes the nontrivial overlaps of modules across datasets. Triangles in this network correspond to approximately conserved modules across all three datasets. Communities in this network (dotted ovals) represent collections of modules that are conserved together and thus have similar biological function. Note that communities in the network can overlap (e.g. module P1 in the schematic belongs to two communities). (**C**) Genes derived from the module communities are called *consensus genes* and were used for downstream bioinformatics analyses including gene ontology enrichment analysis using the g:Profiler tool, testing for intrinsic subset-specificity, and functional interaction network analysis using the IMP functional network. Each of these downstream analyses is independent and complementary.

We analyzed a compendium of three whole transcriptome datasets from SSc skin biopsies (Milano et al. [1], Pendergrass et al. [11], and an expanded version of Hinchcliff et al. [4]; see Materials and Methods). These datasets consist of 70 patients with dSSc, 10 patients with limited SSc (lSSc), 4 morphea samples, and 26 healthy controls (Table 1). Our aim was a comprehensive picture of the gene expression abnormalities in SSc skin and we integrated several publicly available tools with a novel consensus clustering procedure. As demonstrated in Fig. 1, our analysis began with gene coexpression clustering (Fig. 1A), followed by a novel post-processing step called Mutual Information Consensus Clustering (MICC) that identified *conserved* gene expression modules across the three cohorts (Fig. 1B). The outputs from MICC were consensus clusters, i.e. modules that were conserved across datasets, which were the objects of further study, including ontology annotation and functional interaction analysis (Fig. 1C).

**Figure 2:**
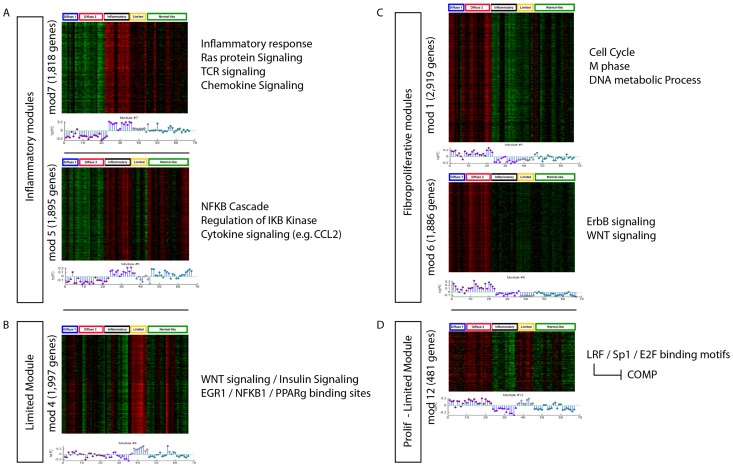
Gene expression modules associated with the intrinsic subsets of SSc. We identified 54 major sets of genes (modules) using WGCNA that define the spectrum of gene expression in SSc skin using Milano as a test case. The top 6 most significant modules are shown and each shows a statistically significant association with the intrinsic subsets (including the limited subset). Module assignment for each gene is unique. The genes that compose the subset-specific modules represent more than 40% of the protein-coding genes in the human genome. Therefore, the intrinsic subsets seem to be determined by a large fraction of the encoded genes. The module eigengene of each module is shown in a stem-plot below each heatmap with intrinsic subsets indicated by color above the heatmap. Proliferative, red; inflammatory, purple; limited, yellow; normal-like, green. (**A**) Inflammatory modules (p<10^−9^ and p<10^−7^; Kruskal-Wallis non-parametric ANOVA corrected for multiple testing), (**B**) Limited Module (p<0.006), (**C**) Fibroproliferative modules (p<10^−7^; p<10^−8^), (**D**) Fibroproliferative and Limited expression module (p<10^−9^). Enriched molecular processes are indicated for each subset to the right of each heat map.

To understand the molecular processes at work in SSc skin biopsies, we constructed data-driven partitions of the expressed genes across multiple SSc skin gene expression datasets using weighted gene co-expression network analysis (WGCNA) [10] (Figs. 1A, 2). Each co-expression cluster, or module, in the partition corresponds to a collection of correlated molecular processes present in the SSc tissue at the time of biopsy. To compare these modules across SSc datasets, we used mutual information to detect when a module from one dataset is present in another dataset. The partitions of the genome-wide expression data vary from one dataset to the next due to clinical heterogeneity and treatment effects, as well as technical variation in RNA processing protocols. All samples were analyzed on Agilent DNA microarrays with the same DNA probes in the same laboratory, providing consistency of the gene expression data and genes analyzed.

**Figure 3:**
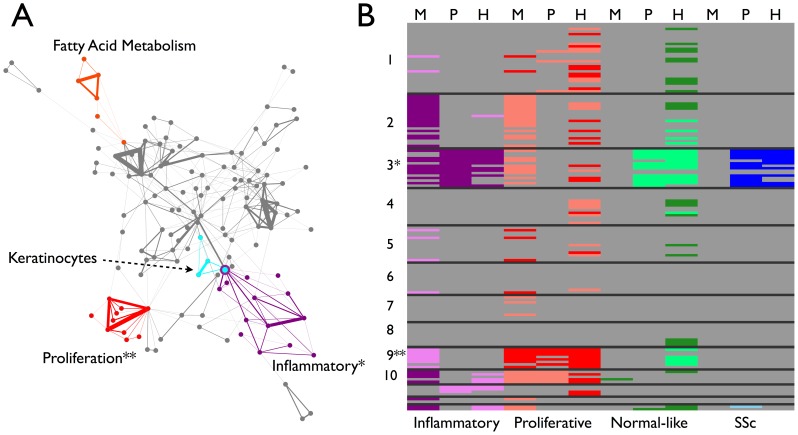
Information graph and consensus clusters for the MPH cohorts. (**A**) The information graph of the MPH cohorts is highly modular (cf. S2 Fig.), indicating approximate conservation of gene expression modules across datasets. The information graph is tripartite by construction, so a triangle in the graph necessarily connects modules across all three datasets. The triangles form communities of mutual edge sharing. Colored nodes and edges highlight four of these communities. The purple community contains modules that are up-regulated in the inflammatory subset (cf. panel B). The red community contains modules that are up-regulated in the fibroproliferative subset (cf. panel B). The cyan community contains modules that are enriched for keratinocyte-specific processes. The orange community contains modules that are enriched for fatty acid metabolism genes. The remaining communities (22 in all and not colored to avoid cluttering the display) are enriched primarily for housekeeping processes and are neither skin- nor disease-specific (see Table 3). (**B**) Modules from the communities were tested for their enrichment in the subsets. Each row corresponds to a triangle in the information graph and each column corresponds to a dataset. The black lines separate communities, e.g. all of the rows in the block marked “1” correspond triangles in community 1. The cells are colored according to whether the module was significantly differentially expressed in a subset with dark colors representing up-regulation and light colors representing down-regulation (Bonferroni-corrected Wilcoxon rank sum p-value p<0.05). We assessed statistical significance of modules within each dataset for each of the three diffuse SSc intrinsic subsets, as well as all SSc vs. healthy controls (Purple- Inflammatory, Red- Proliferation, Green- Normal-like, Blue- All SSc). Note the inflammatory up community (*) and the fibroproliferative up community (**). Note also that community 2 is significantly highly expressed in the inflammatory subset and lowly expressed in the proliferative subset in Milano only. Likewise, community 9 appears to be expressed at low levels in the inflammatory subset in Milano, but none of the other data sets.

To identify genes with conserved expression across multiple datasets, we developed a procedure called Mutual Information Consensus Clustering (MICC) that detects significant conservation of a piece of a module and groups these conserved modules into collections called communities, which are sets of modules with considerable mutual overlap between datasets. Each community is associated with a gene set; namely all genes that are annotated to a module in that community for each dataset. We call these gene sets consensus clusters. The basis of MICC is the concept of mutual information from information theory [14]. Specifically, we use mutual information of partitions (MIP), which is an information measure specific for partitions. MIP quantifies the amount of information one partition has about another; i.e. it measures the correlation of cluster labels across datasets. MICC identifies consensus clusters using MIP to build a module similarity network of significant module overlaps, which we call the information graph (Figs. 1B, 3A). Then MICC algorithmically identifies communities in that network (Fig. 1B; Fig. 3A, colored nodes). These communities are collections of modules that have substantial overlap among each other, and they represent nearly all of the mutual information between the genomic partitions. In this way, MICC extracts almost all of the available information present in the separate clusterings of individual datasets and reports the clusters that are conserved across the three cohorts (see Materials and Methods for a detailed description).

### WGCNA identifies that large numbers of genes in the genome are deregulated in SSc skin

Weighted gene coexpression network analysis (WGCNA) [10] is a gene co-expression clustering procedure that automatically detects the number of modules in a dataset and removes outlier genes. WGCNA performed on a single SSc skin dataset (Milano et al. [1], S1 Data file), demonstrates the complexity of comparative studies across multiple datasets (Fig. 2). The molecular subsets termed ‘SSc intrinsic subsets’ were first identified by Milano et al. [1]. The Milano et al. dataset is the best characterized dataset showing the SSc intrinsic subsets that has been analyzed to date. These data were clustered into modules by WGCNA, and the resulting modules were summarized by their first principal component or module eigengene (Fig. 2). Module eigengenes are a one-dimensional summary of the gene expression within a module that captures the bulk of the variance within that module. To identify those modules that were intrinsic subset-specific, we performed Kruskal-Wallis tests on the module eigengenes with groups defined according to intrinsic subsets. Of the 54 total modules, 23 had a significant p-value for association with the intrinsic subsets (all p<0.05 after Bonferroni correction; Fig. 2 shows six representative examples). These 23 modules comprise approximately 40% of the expressed genes in the genome (Table 2), demonstrating that the intrinsic subsets found in SSc skin are defined by deregulation of a very large fraction of the expressed genes in any given cell. Gene expression for six significant modules along with their corresponding module eigengenes demonstrates clear association with the intrinsic subsets (Fig. 2). These modules are enriched for broad functional categories previously associated with SSc, including chemokine signaling, NFκB signaling, RAS-RAC signaling in the inflammatory subset, and cell cycle processes in the fibroproliferative subset [1], [4], [11].

**Table 2:** Statistics of module conservation.

Dataset	Milano	Pendergrass	Hinchcliff
Number of subset-specific modules (Number of modules total)	17 (54)	13 (66)	31 (58)
Portion subset specific	31%	18%	53%
Number of genes in subset-specific modules	10,373	4,004	8,517
Number of conserved modules	32	50	47
Portion conserved	59%	76%	81%

The number of modules identified by WGCNA varied across datasets. However, a large majority of modules from each dataset were at least partially conserved across the three cohorts, meaning that they were present in at least one triangle of the information graph (Fig. 3A). A smaller fraction of the modules were subset-specific within their dataset. The Hinchcliff dataset had the largest fraction of subset-specific modules, which may be due to subject enrollment in the cohort rather than to SSc biology. The Milano dataset had the largest number of genes present in subset-specific modules. This shows that the subsets are associated with a large number of genes (up to 40% of the genome in Milano et al. assuming an upper bound of 25,000 human genes).

To identify the core set of genes reproducibly found in each SSc intrinsic subset, we performed WGCNA on two additional SSc skin gene expression datasets: Pendergrass et al. [11] and an expanded version of Hinchcliff et al. [4] (Data files S2–S3). The Hinchcliff data are from an ongoing clinical trial of mycophenolate mofetil (MMF). Preliminary data have been published [4], but we also analyzed data for an additional 82 unpublished samples from that trial (the full data available from NCBI GEO at GSE59787). A summary of the three cohorts, including the expanded Hinchcliff cohort, is available in Table 1. Each of the three datasets had approximately 60 modules. The module eigengenes were tested for association with the intrinsic subsets, and it was found that, within a dataset, between 18% and 67% of the modules were subset-specific (Table 2). This shows that for each dataset a substantial fraction of modules was associated with the intrinsic subsets and, that 4,004–10,373 genes out of the approximately 19,500 in the human genome were in differentially regulated modules associated with the intrinsic subsets (Tables 2 and 3).

**Table 3:** Molecular processes enriched in the 13 largest consensus clusters.

Consensus Cluster	Enriched Molecular Processes	# Consensus genes
1	Tubulin processing	1144
2	Insulin signaling	1194
3	TGFβ signaling	312
	PDGF signaling	
	Collagen fibril organization	
	B cell receptor signaling	
	Monocyte chemotaxis	
	Response to interferons	
	Patterning of blood vessels	
4	RNA processing and transport	873
	Ribosome biogenesis	
5	Tubulin processing	485
6	ARF activity	225
7	Mitochondria	73
8	Mitochondria	80
9	Fibrin/fibrinogen	274
	Mitotic spindle	
	G2/M transition	
	Integrin interactions with fibrin	
10	Alcohol metabolism	80
11	Fatty acid metabolism	98
12	DAP12 (TYROBP) signaling	63
	Keratinocyte processes	
13	Phosphatidic acid	11

An analysis with g:Profiler resulted in many biological processes enriched in the each of consensus clusters. This table contains a condensed list of the significant pathways (p<0.05, corrected for multiple testing by default in g:Profiler), retaining those that are specific for skin or other housekeeping biology. Note particularly consensus clusters 3 and 9, which are enriched for the inflammatory and fibroproliferative subsets respectively. Consensus clusters 3, 9 11, and 12 are highlighted in Fig. 3A by the colored communities in the information graph.

The gene co-expression modules represent biological processes that are active in skin and some are reflective of disease pathogenesis. To determine which processes were conserved across all three datasets, we constructed the information graph for the three separate WGCNA partitions of the genome (Fig. 3A). The information graph is a network where a node in the network is a module from one dataset, and a link between modules indicates that the overlap between those modules is significantly larger than would be expected at random. In other words, an edge represents conservation of a significant part of a module across two datasets (see Materials and Methods for a detailed discussion of module overlap scores). Triangles in the information graph correspond to a significant three-way overlap of modules or, equivalently, a module conserved across all three datasets. We enumerated all triangles in the information graph to identify all such conserved modules. There were 157 triangles and approximately 2000 genes in their corresponding triple overlaps. Most (129 out of 178) of the modules across all SSc datasets are present in at least one triangle, i.e. *most* co-expression modules had a significant portion co-expressed in the other datasets (Table 2, bottom row). This indicates that the WGCNA-derived modules are reproducible features of SSc gene expression. Nine of the triangles had all three nodes (modules) significantly associated with the subsets (five inflammatory, four fibroproliferative; see below and Fig. 3).

The consensus genes are hubs in the gene-gene co-expression networks. To see this, we noted that module eigengenes represent hubs in the gene-gene correlation network [15]. A module eigengene does not correspond to an actual gene, but rather represents a theoretical gene that is most central in the module. Therefore, genes that are highly correlated to their module eigengene are more central within their module. We calculated the correlation of each gene to its corresponding module eigengene (S1 Fig.). The density of these gene-eigengene correlations is shown for all genes in the genome (blue curve) and for only the consensus genes (red curve). The consensus genes are significantly more correlated with their module eigengene than randomly selected genes are with their module eigengene, indicating that the consensus genes are significantly enriched for hub genes in their (dataset-specific) co-expression network. This is a useful positive control for the MICC method because it shows that the consensus genes are enriched for “hubness” in the SSc co-expression network and thus MICC finds genes that have salient network features.

### The information graph reveals conserved, subset-specific molecular modules

While most modules are partially conserved between the three datasets and many of them are intrinsic subset-specific, not all intrinsic subset-specific modules are conserved across all datasets (S4 Data file). To find the conserved, intrinsic subset-specific modules, we noted that the information graph has groups of triangles with considerable mutual edge-sharing (Fig. 3A). Many of the triangles in the information graph overlap and form communities of triangles (Fig. 3A, S2 Fig.). This was intriguing because it opened up a broader interpretation of “consensus cluster”. If the information graph had been a disconnected collection of single triangles, this would have implied that there was a one-to-one mapping between the modules from different datasets. Instead, a single module from one dataset gets broken into pieces in the other datasets. The community structure of the information graph indicates what we have known from many prior microarray studies, namely that specific groups of genes are commonly expressed together and that the aggregate set of genes underlying these multiple co-expression clusters constitutes the truly conserved processes in SSc [1], [6], [16].

We detected communities in the information graph using a variant of clique percolation [17], a network community detection procedure that, in this case, explicitly identifies communities of triangles (S1 Text). Clique percolation identified 26 communities, 13 of which were single, isolated triangles, while the rest were groups of more than one triangle (Fig. 3A, S2 Fig.).

To derive a gene set associated with a community in the information graph, we took all modules within the community, computed their union within datasets, and computed their intersection across datasets (S3 Fig.). In this way, we captured all genes whose co-expression was conserved across the three datasets. (A mathematical description of this procedure is presented in the Materials and Methods.) We termed these community-derived gene sets consensus clusters (CCs). Using g:Profiler [18], we found that the consensus clusters are enriched for many biological processes (summary in Table 3; raw data in S5 Data file) present in both healthy and SSc biopsies. For example, CCs 1, 4, 5, 7, 8, and 11 are enriched for basic metabolic and cellular processes, while CC 12 showed enrichment for keratinocyte-specific processes (Table 3; Fig. 3A, cyan). These consensus clusters show that MICC extracts biologically coherent sets of genes that are known to be active in skin as consensus clusters. This provides an additional positive control for the MICC method.

More importantly, CC3 and CC9 showed enrichment for processes implicated in SSc (Table 3; S5 Data file). CC3 was enriched for response to interferons, B cell receptor signaling, monocyte chemotaxis, and TGFβ and PDGF signaling, as well as ECM remodeling processes. CC9 showed enrichment for cell cycle and cell proliferation processes, as well as integrin interactions with fibrin. Note that CC3 and CC9 both show enrichment for distinct ECM-related molecular processes. These data are consistent with the analysis of experimentally derived pathway signatures [19].

The consensus clusters CC3 and CC9 map to the major intrinsic subsets previously described [1]. We tested every module for association with the intrinsic subsets (see Materials and Methods) and we constructed a “heatmap” of the triangles in the information graph by dataset (Fig. 3B). The rows were ordered by community membership and the columns were ordered by dataset. We concatenated each of these plots so all subsets, datasets, and consensus clusters can be viewed simultaneously. Only consensus clusters 3 and 9 were enriched for SSc intrinsic subset specificity. Consensus cluster 3 contained modules that are almost all significantly expressed at high levels in the inflammatory group of patients (Fig. 3A, purple nodes; Fig. 3B). Consensus cluster 9 contains modules that are almost all significantly expressed at high levels in the fibroproliferative group (Fig. 3A, red nodes; Fig. 3B). We also included tests for all SSc biopsies versus healthy controls to determine if there were any consensus clusters that were generally conserved across all SSc biopsies. There were no consensus clusters that were enriched for all SSc versus healthy controls, which illustrates quantitatively SSc heterogeneity. Furthermore, there were no consensus clusters that were consistently expressed at low levels in any of the subsets.

Some consensus clusters are enriched for a subset in some of the datasets, but are not replicated across all datasets (Fig. 3B). For example, CC2 is expressed at high levels in the inflammatory subset and low levels in the proliferative subset in Milano, but neither of the other datasets. Inflammatory-specific CC3 is expressed at low levels in the proliferation subset in Milano and in the normal-like subset in Pendergrass and Hinchcliff, and is expressed at high levels in all SSc versus healthy controls in Pendergrass and Hinchcliff only. Similarly, CC9, which is proliferative-specific, is expressed at low levels in the inflammatory subset in Milano only. These observations demonstrate that genes with increased expression should be the focus in SSc.

### Conserved inflammatory and fibrosis genes form a network with putative SSc risk alleles

**Figure 4:**
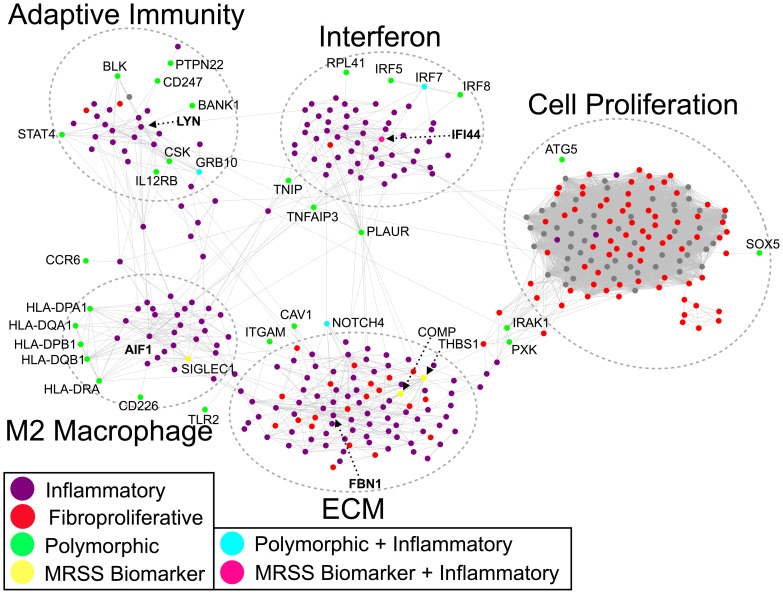
Molecular network of inflammatory and fibroproliferative consensus genes. The consensus genes for the inflammatory and fibroproliferative subsets are connected in the IMP functional network. Inflammatory genes are colored purple, while fibroproliferative genes are colored red. Genes with polymorphisms are colored in green and MRSS biomarker genes are colored yellow. One MRSS biomarker gene (*IFI44*) was also an inflammatory consensus gene (pink), while three polymorphic genes were inflammatory consensus genes (turquoise). Note the five distinct subnetworks corresponding to type I interferons, M2 macrophages, ECM proteins and TGFβ signaling, adaptive immunity, and cell proliferation. The interferon, M2 macrophage, and adaptive immunity subnetworks are composed almost exclusively of inflammatory genes, while the ECM subnetwork shares genes from both intrinsic subsets. Furthermore, the polymorphic genes interact primarily with inflammatory subset genes indicating that the genetic risk in SSc is related to immune abnormalities.

The biology of CC3 and CC9 show the processes common to the intrinsic subsets that have been observed across multiple gene expression datasets: inflammation, cell interactions with ECM, and cell proliferation (Table 3). To determine if there was a more interconnected relationship between these conserved processes (such as genes related to specific cell types) than could be gained from an ontological annotation analysis like g:Profiler, we used CC3 and CC9 as a query gene set for the IMP gene-gene interaction Bayesian network (IMP) (Fig. 4) [20]. IMP is a gene-gene interaction network developed using a large compendium of high-throughput biological data including all publicly available microarray data that predicts the probability that pairs of genes have a co-expression interaction. A list of genes is imported into IMP, and a list of high-probability interactions between the genes on the imported list and (up to 50 additional genes in) the rest of the genome is generated. IMP is completely agnostic to SSc-specific biology and reports predicted interactions that are based on the preponderance of evidence across all publicly available gene expression data. As our query, we pooled the two consensus clusters CC3 and CC9 to discover possible molecular links between the inflammatory and fibroproliferative intrinsic subsets. We added polymorphic genes from genome-wide association studies (GWAS), as well as genes from candidate gene studies that have been replicated in at least one follow up study (see Materials and Methods; S6 Data file). In addition, we added four genes that are putative predictors of Modified Rodnan Skin Score (MRSS), a widely used clinical measure of skin fibrosis [5].

The output network from IMP was dominated by one large interconnected network that had five distinct subnetworks (Fig. 4; S7–S9 Data files). The five molecular subnetworks were each enriched for a distinct biological process: interferon response, M2 macrophage activation, adaptive immunity, ECM deposition and remodeling and TGFβ signaling, and cell proliferation.

One subnetwork was dominated by interferons and interferon-inducible genes (Fig. 4, top middle; S9 Data file). The interferon subnetwork contained genes solely from the inflammatory consensus cluster (Fig. 4, purple nodes). This subnetwork contained the interferon inducible genes *IFI16* and *IFI44*, the latter of which is a putative biomarker of fibrosis [5]. This subnetwork also contains the polymorphic interferon regulatory factor genes *IRF5*, *IRF7*, and *IRF8*.

A second subnetwork contained genes characteristic of M2 macrophage activation (Fig. 4, bottom left; S9 Data file). The genes in this network, which include major histocompatibility complex (MHC) class II genes with SSc-associated polymorphisms, are derived primarily from the inflammatory consensus cluster, implicating macrophages as mediators of inflammation. Polarized macrophages can broadly be categorized as “classically activated” (M1) or “alternatively activated” (M2), although it is important to recognize that macrophage polarization encompasses a broad spectrum of activation states. M1 macrophages may be elicited through stimulation with IFN-γ and LPS, are microbicidal, and promote Th1-mediated immune responses. In contrast, M2 cells, which mediate immune suppression, may be activated by various stimuli, including IL-4 and/or IL-13, which are elevated in SSc sera [21], [22]. Genes associated with M2 activation, including *CX3CR1* [23], *IL10R* [24], and *HLA-DMB* [25], were consistently expressed in this subnetwork, in accordance with previous studies that found increased M2-polarized macrophages in SSc skin compared to healthy skin [26]. As M2-polarized cells regulate vascularization and are a potent source of TGFβ, PDGF, and inflammatory cytokines [27]–[29], activated M2 macrophages may play a role in mediating fibrosis and inflammation in SSc.

A third molecular subnetwork contained genes related to adaptive immunity (Fig. 4, top left; S9 Data file). There are relationships to both B and T cells in the genes in this subnetwork. Two chains of the T cell receptor complex are represented: *CD3G* (gamma chain of the T cell receptor (CD3)) and *CD247* (the zeta chain of the T cell receptor), which contains SSc-associated polymorphisms. The IL-12 pathway, which mediates Th1 cell differentiation and activation [30], [31], is represented through *IL12RB2*. Binding of IL-12 to IL12RB2 on activated T cells initiates a signal transduction cascade that results in activation of STAT transcription factors, including STAT4 [32] (also represented in this subnetwork), which regulate T cell signaling and immune activation [33]. Aberrant expression of *IL12RB2* has been reported in autoimmune and infectious diseases [34], [35], implicating this gene as an important regulator of inflammation and immune defense.

B cell receptor activation and signaling are also represented in this subnetwork. *DOCK10* expression is up-regulated in B cells by pro-inflammatory IL-4 [36], and *BANK1* and *BLK* are B cell proteins that have polymorphisms associated with SSc. Both *LYN* and *CSK* appear in this subnetwork and are directly connected to each other. The tyrosine kinase LYN, which plays a critical role in down-regulating B cell activation and mediating self-tolerance [37], [38], is phosphorylated by CSK [39]. Polymorphisms in CSK have been linked to both SSc and systemic lupus erythematosus (SLE) and are associated with aberrant B cell signaling [40]. CSK also associates with Lyp [41], which is the product of the tyrosine phosphatase PTPN22. The *PTPN22* gene also contains an SSc-associated polymorphism. Mutations in *PTPN22* that interfere with its ability to bind to CSK also interfere with both B and T cell receptor activation [42], [43]. Moreover, mutations in *PTPN22* have been reported in a variety of other autoimmune diseases, including SLE, rheumatoid arthritis, and type 1 diabetes [44].

Negative regulators of B and T cell activation such as *SOCS2* and *SOCS3*, are included in this network. SOCS3 has been shown to directly inhibit IL-12-induced STAT4 activation [45]. The co-occurrence of pro- and anti-inflammatory signals in this subnetwork is notable and is likely because our data are derived from whole skin biopsies (see Discussion).

The fourth molecular subnetwork contained TGFβ pathway genes (which have long been implicated in the activation of fibrosis in SSc [46], [47]) and ECM structural proteins (Fig. 4, bottom middle). This TGFβ/ECM subnetwork contained genes from both the inflammatory and fibroproliferative consensus clusters (Fig. 4, red and purple nodes; S9 Data file). We also found expression of genes associated with Notch signaling such as *NOTCH4*, which contains SSc-associated polymorphisms, and with the epithelial-mesenchymal transition (EMT) such as *LATS2*. Alternatively activated macrophages are known to produce large quantities of TGFβ in SSc pulmonary fibrosis [29], suggesting that the M2 macrophage subnetwork could drive activation of the TGFβ/ECM subnetwork.

The final molecular subnetwork contained cell cycle/cell proliferation genes, which were primarily from the fibroproliferative consensus cluster (Fig. 4, right). The expression of proliferation genes is commonly observed in cancer [2], [7], [48] and their presence in the gene expression data of SSc was a surprising and unexpected finding [1]. The large and densely interconnected subnetwork of genes in Fig. 4 (right, red nodes) was composed almost exclusively of cell cycle-regulated genes including *AURKA/B*, *CCNA2*, *CCNB1*, *CHK1*, and *DHFR* [7]. This subnetwork was conserved and showed increased expression in the fibroproliferative subset of patients across all three cohorts, and constituted the core gene expression signature in that subset of patients (Fig. 4, red nodes). Therefore, the cell proliferation signature of the fibroproliferative subset of patients first observed in Milano et al. [1] is a conserved feature of SSc across three independent cohorts from three separate clinical centers. This molecular subnetwork has connections to each of the other four subnetworks (interferon, M2 macrophages, adaptive immunity, and TGFβ/ECM) suggesting that cell proliferation in SSc skin is modulated by the inflammatory and ECM remodeling processes in skin.

IMP predicts that the genes linked to SSc-associated polymorphisms (30/41 total) and the putative MRSS biomarker genes of Lafyatis and co-workers (4/4) have interactions within this large component of the molecular network (Fig. 4). Polymorphisms in *IRF5*, *IRF7*, and *IRF8* were linked to the interferon subnetwork. *IRF7* is also differentially expressed in the inflammatory subset. The polymorphisms associated with human leukocyte antigen (HLA) alleles predominantly have interactions with the M2 macrophage subnetwork of genes. Polymorphisms in and differential expression of *NOTCH4* were linked to the TGFβ/ECM subnetwork. The same was true for the MRSS biomarker genes; *IFI44* was linked to the interferon subnetwork; *SIGLEC1* was linked to the M2 macrophage subnetwork; and both *COMP* and *THBS1* were linked to the TGFβ/ECM subnetwork. These results suggest that prediction of worsening skin disease requires sampling genes from each molecular subnetwork.

### The molecular network contains SSc-pathology-specific hubs

The molecular network contains genes that are hubs (i.e. highly connected nodes) of the subnetworks.

**Figure 5:**
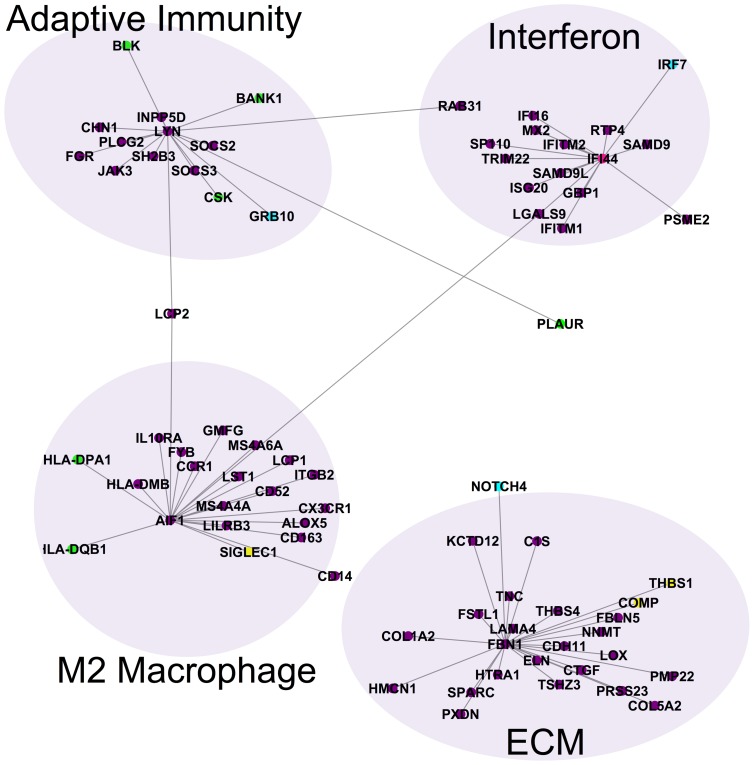
Hubs in the inflammatory and ECM components of the network. The putative MRSS biomarker gene *IFI44* is a hub of the type 1 interferon subnetwork. *AIF1*, which contains SSc-associated polymorphisms and is related to M2 macrophage polarization, is a hub of the M2 macrophage network. *FBN1*, which contains SSc-associated polymorphisms in some populations and is a key component of ECM that regulates matrix stiffness, is a hub of the TGFβ/ECM network. The tyrosine kinase gene *LYN* is associated with B cell activation and mediating self-tolerance and is a hub in the adaptive immunity subnetwork.

Interferon-induced protein 44 (IFI44) is a hub of the interferon subnetwork. It has conserved high expression across all three of our cohorts in the inflammatory subset and is one of the most highly connected genes in the interferon subnetwork (Fig. 5, top right). *IFI44* is predicted to have co-expression interactions with several other interferon-inducible and interferon-regulating genes, including *IFI16*, *IRF7*, *IFITM2*, *ISG20*, *GBP1*, and *TRIM22*.

Allograft Inflammatory Factor 1 (AIF-1) is a hub of the M2 macrophage. *AIF1* is consistently highly expressed in the inflammatory subset across all three SSc skin cohorts and is one of the most highly connected genes in the M2 macrophage subnetwork (Fig. 5, bottom left). In the molecular network (Fig. 5, bottom left), *AIF1* has many links including: *ITGB2*, a binding partner of the monocyte marker *ITGAL*, and MHC class II genes *HLA-DMB*, *HLA-DPA1*, and *HLA-DQB1*. In addition, *AIF1* has connections to chemokine receptors *CCR1* and *CX3CR1*, which are connected to chemokines *CX3CL1* (fractalkine) and *CCL2* (MCP-1).

The tyrosine kinase gene *LYN* is a hub of the adaptive immunity subnetwork (Fig. 5, top left). *LYN* has predicted edges with four polymorphic genes in this subnetwork: *BLK*, *BANK1*, *CSK*, and *GRB10*. *LYN* also has connections to the polymorphic, bridge genes *PLAUR* and *LCP2* (see below), and suppressors of cytokine signaling genes *SOCS2* and *SOCS3*. The conserved finding of high expression of *LYN* in the inflammatory subset and its centrality within the adaptive immune subnetwork suggests that *LYN* plays a key role in the adaptive immune component of SSc in skin.

Fibrillin-1 (FBN1) is a hub of the TGFβ/ECM subnetwork (Fig. 5, bottom right). High expression of *FBN1* is conserved across the inflammatory subset of all three cohorts of SSc skin, and *FBN1* is highly connected within the TGFβ/ECM subnetwork of the molecular network (Fig. 5, bottom right). The TGFβ/ECM subnetwork includes genes that primarily show high expression in the inflammatory subset but also includes genes that are highly expressed in the fibroproliferative group, thus providing a putative molecular link between the two groups. *FBN1* has predicted connections to many genes whose increased expression is conserved, including: pro-fibrotic genes including *COL1A2*, *COL5A2*, and elastin (*ELN*), *CTGF*, *SPARC*, *THBS1*, *THBS4*, *COMP*, *TNC* and ECM remodeling and wound response genes *LOX*, *NNMT*, and *FBLN5*. In addition, *FBN1* has connections with growth factor genes and receptors such as *HTRA1* and *NOTCH4*; cell adhesion genes *CDH11* and *LAMA4*; as well as the complement system gene *C1S*.

### The molecular network shows genes that bridge subnetworks

**Figure 6:**
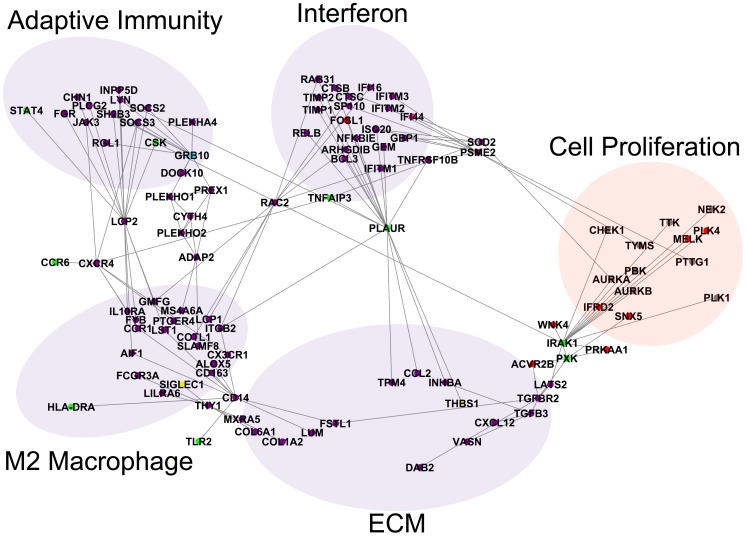
Bridges between components of the network. Several genes bridge the component subnetworks of the molecular network. *PLAUR* is a gene that contains SSc-associated polymorphisms that forms a bridge between the interferon subnetwork and TGFβ/ECM subnetwork. The gene *RAC2* is a bridge between the interferon and M2 macrophage subnetworks. The genes *LCP2* and *CXCR4* are bridges between the M2 macrophage subnetwork and the adaptive immunity subnetwork. There are also several paths through *GRB10* to *ADAP2* between the M2 macrophage subnetwork and the adaptive immunity subnetwork. The genes *CD14* and *THY1* (*CD90*) are bridges between the M2 macrophage subnetwork and the TGFβ/ECM subnetwork. The genes *IRAK1* and *PXK* are bridges between the TGFβ/ECM subnetwork and the cell proliferation subnetwork.

In addition to containing discrete subnetworks, the molecular network also shows genes that bridge the subnetworks (Fig. 6). These genes are of particular interest because they have predicted connections between multiple, distinct subnetworks. The primary reason for using CC3 and CC9 simultaneously as queries to IMP was to identify possible molecular connections between the core molecular processes of the inflammatory and fibroproliferative intrinsic subsets. The bridge genes live at the interfaces between the subnetworks that constitute these core molecular processes.

The genes *CXCR4* and *LCP2* are the major connections between the adaptive immunity subnetwork and the M2 macrophage subnetwork (Fig. 6). *LCP2* (*SLP*-76), which modulates T cell activation [49], has predicted interactions with *AIF1* and *IL10RA* in the M2 macrophage subnetwork and to *SOCS2*, *SOCS3*, *STAT4*, *LYN*, and *CSK* in the adaptive immunity subnetwork (see edges extending from LCP2 in Fig. 6). The chemokine *CXCR4* has predicted interactions with the cytokines/chemokines *IL10RA*, *CX3CR1*, *CCR1*, and the polymorphic *CCR6* in the M2 macrophage subnetwork (Fig. 6). *CXCR4* has predicted interactions with *SOCS3* and *JAK3* in the adaptive immunity subnetwork.

*GRB10* contains an SSc-associated polymorphism and is also expressed at high levels in the inflammatory subset (see blue *GRB10* node, Fig. 6). *GRB10* is part of a complex path from the adaptive immune subnetwork to the M2 macrophage subnetwork that includes genes containing pleckstrin homology domains including *PLEKHO1*, *PLEKHO2*, *CYTH4* and *ADAP2*.

The major connection between the M2 macrophage subnetwork hub *AIF1* and the interferon subnetwork hub *IFI44* is through *RAC2*. *RAC2* encodes a member of the Rac family of signaling molecules and has multiple predicted interactions with both the interferon subnetwork and the M2 macrophage subnetwork (Fig. 6). In the interferon subnetwork (Fig. 6, upper middle), *RAC2* connects to *CTSC* (cathepsin C), *IFITM1* and *IFI16*, as well as the Rho GTPase related genes *ARHGDIB* and *RAB31*. In the M2 macrophage subnetwork (Fig. 6, lower left), *RAC2* connects to *ITGB2*, the actin cytoskeleton related proteins *LCP1* and *COTL1*, and *GMFG*. *COTL1* is also related to leukotriene biosynthesis through a known interaction with *ALOX5*. These diverse interactions suggest that *RAC2* is involved simultaneously in macrophage motility, leukotriene biosynthesis, and interferon signaling.

The major bridges between the M2 macrophage subnetwork and the ECM subnetwork are *THY1* (*CD90*) and *CD14* (Fig. 6, lower left). *THY1* connects to *SIGLEC1*, *MXRA5* and *COL1A2*. *THY1* mediates adhesion of leukocytes and monocytes to endothelial cells and fibroblasts [50], may also have a role in lung fibrosis (a major complication of SSc); *THY1* knockout mice have increased lung fibrosis [51], [52]. *CD14* is a cell surface protein mainly expressed by macrophages, is inducible by and connected to *AIF1* [53]. It also has connections to the polymorphic genes *TLR2* and *HLA-DRA* (Fig. 6, lower left).

*PLAUR* (*UPAR*) contains a putative SSc-associated polymorphism, is a member of the interferon subnetwork, and has numerous links with the ECM, M2 macrophage, and the adaptive immunity subnetworks (Fig. 6). *PLAUR* encodes the plasminogen activator, urokinase receptor protein and is a pleiotropic gene at the interface of ECM remodeling, as a component of the fibrinolysis system, and in both adaptive and innate immune processes, including monocyte migration [54]. *PLAUR* is inducible by proinflammatory cytokines IL1β and TNFα. *PLAUR* connects to the tyrosine kinase *LYN*, the hub gene of the adaptive immunity subnetwork, and to the integrin gene *ITGB2* in the M2 macrophage subnetwork. It is also connected to the polymorphic genes *TNFSF10B* and *TNFAIP* in the interferon network and to *TPM4*, *INHBA*, *THBS1*, and *CCL2* in the ECM subnetwork. The centrality of *PLAUR* within the consensus gene network suggests that *PLAUR* may be a key mediator of inflammatory and ECM remodeling signals in SSc skin.

The proliferation subnetwork has predicted interactions with the inflammatory and ECM subnetworks. The most pronounced connection is between the ECM subnetwork and the cell proliferation subnetwork through TGFβ pathway genes (Fig. 6). The TGFβ pathway is known to modulate cell proliferation. There are multiple paths from the TGFβ pathway genes *TGFB3* and *TGFBR2* to the cell proliferation subnetwork through the polymorphic genes *IRAK1* and *PXK,* which have predicted interactions with the serine/threonine kinases *LATS2*, *WNK4*, and *PRKAA1*. Serine/threonine kinases are well known to be important regulators of cell proliferation and they are bridges between the ECM subnetwork and cell proliferation network.

## Discussion

The intrinsic subsets of SSc have been found in multiple skin gene expression datasets. Until now, the majority of experimental data has indicated that the subsets are mutually exclusive—i.e. patients are categorized as being in one of the subsets, and that the core molecular processes and subsets of genes, are reproducible across cohorts. Despite this consistency, the exact set of intrinsic genes varies across datasets. We address both of these issues here. Our consensus clustering approach allowed us to detect a conserved set of genes from a module perspective across the three independent SSc patient cohorts by considering molecular processes first and constituent genes second. The predicted co-expression interactions between these consensus genes indicate that the key processes represented by the consensus genes (inflammation, ECM remodeling, and cell proliferation) may interact at a molecular level, with specific links between the subnetworks. Thus, we have demonstrated theoretical *connections* between the genes of the SSc intrinsic subsets that are difficult to capture experimentally.

It is clear that in addition to its clinical heterogeneity, SSc is a genetically complex disease. Many risk alleles for SSc have been identified, but each has only a modest odds ratio and the complete picture of SSc will likely develop from the *interactions* between various risk factors. The network of consensus genes demonstrates that a significant fraction of the genes with risk alleles for SSc have probable interactions with the consensus genes that underlie the intrinsic gene expression subsets. This implicates these polymorphic genes as interacting with genes differentially expressed in the subsets. This simultaneously provides a picture of the key gene expression abnormalities in the intrinsic subsets and the validated genetic associations at a systems level.

These data and the resulting network were developed from a detailed meta-analysis of SSc skin gene expression datasets using MICC, a consensus clustering framework we developed. Our method reports only consensus clusters that are conserved across all input datasets and dispenses with non-conserved gene expression. The concept of mutual information gives MICC a theoretical foundation, but like any data mining algorithm, its value is gauged by performance on real data.

The rationale for gene coexpression clustering algorithms like WGCNA is that co-expression networks are inherently modular and that co-expression hub genes are likely related to the regulation of the modules. This has been borne out by several studies in humans [55], mice [56], and even across species [57]. The genes identified by MICC are disproportionately more hub-like than a random population of the same size (S1 Fig.). Therefore, MICC does not identify spurious overlaps but rather detects network-relevant overlaps that are enriched for key hub genes. At the same time, the information graph used by MICC is not simply a disconnected set of triangles, which would indicate a one-to-one mapping of modules between datasets. Instead, the modules in one dataset are broken into a small set of pieces that are re-assorted to build the modules in another dataset. This is likely due to variations in study design and protocols between the datasets, but also the inherent heterogeneity of SSc, therapy effects, and environmental exposures. The MICC method is explicitly designed to handle this unavoidable variance by broadening the definition of consensus cluster to allow for imperfect conservation of gene coexpression. We also note that MICC is completely general with respect to the data that are clustered and which clustering algorithms are used. In principle, gene expression from different tissues (e.g. blood and skin) or different species (e.g. mouse and human) or data from multiple experimental modalities (e.g. transcriptomics and proteomics) can be compared using MICC. These types of data exist for multiple tissues in SSc and multiple animal models of SSc. Follow-up studies will integrate these to further elaborate the molecular underpinnings of SSc.

The consensus clusters from MICC show both skin-specific processes that represent basic biological processes in this tissue as well as disease-specific processes. Nearly all (24 out of 26) consensus clusters are enriched only for general cellular or otherwise skin-specific biology: metabolism, cell turnover, keratinocyte-specific gene expression, etc. We view these consensus clusters as a useful positive control for the MICC method. Such housekeeping processes are clearly biologically relevant and MICC would be missing important structure in the data if these were not found. By taking a “module first” approach, MICC is able to identify consensus genes that are specifically clustered into pathologically active modules (the subset-specific modules).

### The MICC-derived consensus clusters are enriched for known mediators of SSc pathology and genetic risk factors

Two of the consensus clusters were SSc subset-specific (Fig. 3B). These clusters contain the key gene expression abnormalities in SSc that are conserved across all three cohorts. The consensus clusters are enriched for inflammatory process Gene Ontology terms, as well as TGFβ signaling, PDGF signaling, and cell proliferation (Table 3). Most (30 out of 41) of the genes with replicated SSc-associated polymorphisms are predicted to interact with genes in the consensus clusters; 28 out of 30 of these interact in the immune (interferon, M2 macrophage, and adaptive immunity) and TGFβ/ECM subnetworks (Fig. 4). The inflammatory-specific consensus cluster also contains the genes *FBN1* and *AIF1*. Previous work implicates *FBN1* in SSc pathogenesis, as a duplication of *FBN1* causes fibrosis in the Tsk1 mouse [58] and a point mutation in *FBN1* causes the fibrotic phenotype in the Stiff Skin Syndrome mouse [59]. Fibrillin-1 forms a matrix of elastic microfibrils that provide a scaffold for elastins and collagens, and a means for sequestering matricellular growth factors. Mouse embryonic fibroblasts expressing the Tsk1 mutant *FBN1* have altered microfibril morphology that results in increased collagen deposition [58]. While polymorphisms in *FBN1* might cause dosage effects that result in fibrosis in some models (e.g. Tsk1), it is possible that chronic inflammation causes chronic high expression of *FBN1* to similar effect in humans. Rare polymorphisms in *FBN1* have been associated with SSc in some subpopulations [60]–[62].

Similarly, *AIF1* is implicated in SSc disease progression. A SNP in *AIF1* has been implicated in anticentromere antibody (ACA) positive SSc [63]. Moreover, AIF-1 is interferon-inducible, constitutively expressed in macrophages [64], and plays a role in vasculogenesis and endothelial cell proliferation and migration [65]. In the Sclerodermatous Graft-Versus-Host Disease (sclGVHD) mouse model of SSc, *AIF1* was found to be highly expressed in skin [66] and to induce fibroblast and monocyte chemotaxis [53]. *AIF1* has many predicted interactions with chemokine receptors *CCR1* and *CX3CR1*, which are connected to chemokines *CX3CL1* (fractalkine) and *CCL2* (MCP-1). The genes *CX3CL1* and *CCL2* are M1 and M2 macrophage-related genes respectively [67] and are chemotactic for monocytes, macrophages, and T cells [68], suggesting enhanced recruitment of inflammatory cells to this subnetwork. A recent study of a mouse model of SSc demonstrated that both *CCR2* and *CX3CR1* regulate skin fibrosis, further implicating these mediators in the pathogenesis of SSc [69]. In addition, *CCL2* has been shown to induce M2 macrophage polarization [70], which may result in persistent M2 activation. The repeated and conserved finding of high AIF-1 levels in the inflammatory subset and its tight connection to innate immune mediators of inflammation suggest it may be involved in enhanced macrophage chemotaxis and activation in SSc skin.

*LYN*, a hub of the adaptive immunity subnetwork, modulates B cell activation and plays a role in self-tolerance. B cell signaling has been implicated in SSc development and progression, as B cells have been shown to play a role in both the development of autoantibodies and cutaneous fibrosis in the Tight Skin 1 (Tsk1) mouse model of SSc. Notably, LYN is overactive in response to overexpression of *CD19* in this model [71]. Thus, *LYN* may play a role in the autoimmune component of SSc in human patients.

### The molecular network shows putative connections between subnetworks

The consensus gene network (Figs. 4 and 6) also implicates genes as bridges *between* the subnetworks. These notably include the polymorphic genes *PLAUR*, *IRAK1*, *PXK*, and *GRB10*. In addition, we find differentially expressed genes straddling the subnetworks including *RAC2* and *LCP2*. The interconnections between the subnetworks present possible molecular paths through which these processes interact.

**Figure 7:**
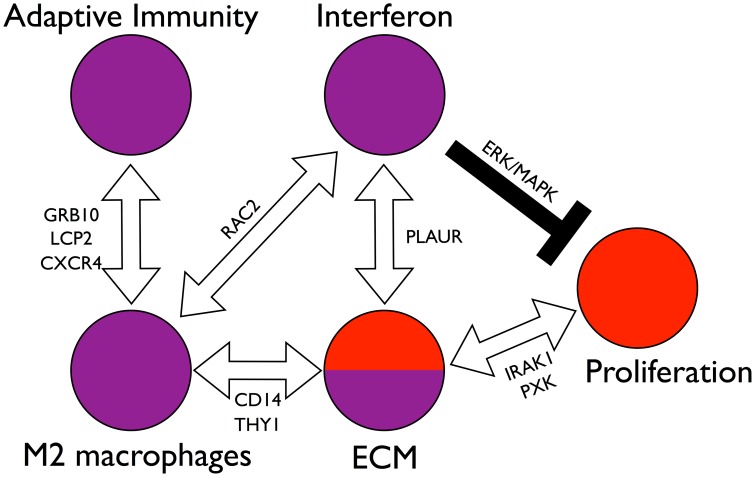
Model of interactions among the components of the network. The molecular network of Fig. 4 is densely interconnected, implicating many possible interactions between the core molecular processes (interferon activation, M2 macrophage activation, adaptive immunity, ECM remodeling, and cell proliferation). Stepping back from the granular detail of single genes, we see a system of distinct parts through which SSc could be initiated and maintained. Among these are paths of particular interest. The interferon subnetwork and the M2 macrophage subnetwork are connected by RAC2. The M2 macrophage subnetwork in turn is connected to the ECM subnetwork through paths through *CD14* and *THY1*. Suggesting macrophages may influence or drive ECM abnormalities in skin. The interferon subnetwork and the ECM subnetwork are connected through paths containing the pleiotropic and polymorphic gene *PLAUR*. The M2 macrophage subnetwork is connected to the adaptive immunity subnetwork through several distinct sets of paths through the genes *GRB10*, *LCP2*, and *CXCR4*. The ECM subnetwork is connected to the cell proliferation cluster through TGFβ pathway genes and paths containing the polymorphic genes *IRAK1* and *PXK*, which suggests that ECM remodeling modulates cell proliferation through the TGFβ pathway. The interferon node may negatively regulate proliferation via the ERK/MAPK pathway resulting in the general mutual exclusivity of the inflammatory and fibroproliferative subsets. Thus we see a set of interconnected, balancing feedback loops that can enforce subset homeostasis, but also allow for patients to transition between the subsets, possibly in response to therapy.

The finding that most SSc-associated polymorphisms are associated with immune system mediators suggests that the initial events in SSc are likely to be immune-regulated and to involve interferon activation (Fig. 7). The immune response in SSc likely differs from a normal response because of predisposing genetic variants in these and associated genes. This may lead to the secondary recruitment of macrophages via RAS-RAC signaling (Fig. 7).

We predict that the interferon network suppresses cell proliferation, given the clear distinction between the inflammatory and fibroproliferative subgroups. This inference is based on known interferon biology and not on the network itself. In contrast, it is possible that the ECM network stimulates cell proliferation through the TGFβ pathway and serine/threonine kinases *IRAK1*, *LATS2*, *WNK4*, and *PRKAA1*. In this model, inflammatory gene expression creates a balancing feedback loop that modulates fibroproliferative gene expression (Fig. 7).

A major strength of the IMP network and its data integration capabilities derives from its ability to provide a more detailed picture of SSc development and progression compared with more conventional approaches. For example, while all of the purple nodes in Fig. 4 are highly expressed in the inflammatory group across all data sets, the IMP network provides information regarding gene-gene interactions in addition to expression data. In this example, the IMP network indicates which subnetworks correspond to discrete processes (interferon, M2 macrophages, ECM, and adaptive immunity) and which interactions are *mediated through the network*. Thus, we gain insight by recognizing that the interferon component is distinct from the M2 macrophage component, despite their co-expression and known interdependence. The value of the IMP network is as much in the connections that are *not present* as those that are.

### What is the nature of the interactions between the intrinsic gene expression subsets?

Since the original publication of the intrinsic subsets, two important questions have been central to their interpretation and their clinical relevance: First, can a patient's subset change over the course of their disease? And second, can the subsets predict therapeutic response?

Pendergrass et al. [11] demonstrated that a patient's subset is stable over time scales of 6 to 12 months. This means either that patients never change subsets and the intrinsic subsets are effectively distinct diseases, or that the subsets are long-lived states of the same disease. Our analysis shows that the inflammatory and fibroproliferative subsets share a molecular network containing TGFβ pathway genes and ECM component genes, suggesting that inflammatory patients may transition to the fibroproliferative subset, perhaps in response to successful immunosuppressive therapy. Indeed, immunosuppressive therapy has not been widely successful for treatment of SSc [72]. On the other hand, fibroproliferative biopsies still have some activation of the TGFβ/ECM network *despite* the absence of the inflammatory signature (Fig. 4). The connection of the subsets through the TGFβ/ECM subnetwork indicates that the fibroproliferative subset shares a common pathway with the inflammatory subset and that the fibroproliferative subset is tied to chronic TGFβ activation and ECM deposition. Thus, based on the molecular network, it is possible that immunosuppressive therapy can move patients to the fibroproliferative subset rather than restoring their gene expression to that of healthy skin. Our data from an ongoing MMF clinical trial and analysis of mouse models of SSc suggests that gene expression changes precede clinical changes [4], [66]; therefore gene expression could act as a readout for the effectiveness of a drug. This idea should be rigorously tested in clinical trials that carefully monitor gene expression in patient skin biopsies.

The pathogenesis of SSc has been enigmatic, but a number of genetic risk factors have been identified by genome-wide association studies and candidate gene studies. Three of these polymorphic genes, *NOTCH4*, *IRF7*, and *GRB10*, are in the inflammatory consensus cluster, and hence are consistently differentially expressed in the inflammatory subset (Fig. 4). This suggests that these may be *cis*-acting alleles and demonstrates the need for candidate gene studies to determine if differential expression is genetically driven in a subset of patients. The IMP functional network predicts that twenty-five of the remaining forty-one polymorphic genes interact with genes from the inflammatory consensus cluster (Fig. 4). Rather than being scattered evenly across all of the subsets or unrelated to any of the consensus genes, the risk alleles are overwhelmingly related to the inflammatory subset. The genetic studies, however, did not stratify their patients by intrinsic gene expression subset. The studies were carried out as case versus control or case versus case, when stratified by autoantibody status or other clinical outcomes. Risk alleles associated with a particular gene expression subset have not been reported. We reemphasize the fact that we found no consensus clusters that were differentially regulated in all SSc vs. healthy control biopsies.

These data support the hypothesis that the subsets are related to disease progression and that SSc starts with immune activation, perhaps in response to an environmental trigger [73], [74]. The SNPs associated with SSc would then likely be risk factors for an aberrant immune response to this trigger. Should such a model be correct, we are still left with the question of why we have different subsets that generally show little or no correlation with disease duration. The simplest explanation for this result is that patients progress through the gene expression subsets at dramatically different rates and that our measures of disease duration are currently inadequate.

Another possibility is that any given patient transitions between these intrinsic gene expression groups in a dynamic manner that we do not observe using serial skin biopsies across 6–12 month time interval. This would mean that cross-sectional studies of patients would still capture all subsets while maintaining a weak correlation to disease duration. We think this is unlikely because serial biopsies are generally found in the same subset.

The final possibility is that the subset a patient stays in, and the duration in which they remain, is dependent on many outside and as yet poorly characterized factors. These could include environmental stimuli that trigger an inflammatory response, or genetic factors that determine the rate at which one progresses through the mechanistic stages of SSc. It is possible that patients in each intrinsic subset have a different set of predisposing genetic polymorphisms or similar environmental triggers. This can only be addressed if we can look for genetic risk factors in a cohort of patients stratified by gene expression subset for genetic risk alleles. There may be genetic risk factors that cause a patient to “stall” at particular point along the progression from inflammatory to proliferative to normal-like. Genetic modifiers of the molecular links in the consensus gene network (Fig. 4) might hold the key to showing why many patients go into spontaneous remission while others experience rapid clinical progression, and indeed, our network analysis suggests candidates for explaining this (Figs. 6, 7). For example, *IRAK1* and *PXK* are polymorphic genes that exist on paths in the network between the TGFβ/ECM network and the cell proliferation network. This strongly argues for future studies that test their possible roles in TGFβ-modulated cell proliferation, with particular attention to their roles in influencing other serine-threonine kinases that modulate the cell cycle.

### Associations with autoantibodies

The presence of antinuclear autoantibodies in patient serum is a widely used biomarker of SSc. To date the intrinsic subsets have shown no clear association with autoantibody status [1], [4], [11], which is consistent with a model by which the subsets represent disease progression. Several genetic polymorphisms are associated with autoantibody status (S9 Data file), including *BLK* and *BANK1*, which are related to ACA- and ATA-positive SSc respectively. These B cell proteins are already attractive candidates for autoantibody production, as they are directly associated with the cells that produce the antibodies, but our network analysis also shows that they are functionally related to adaptive immune genes that are highly expressed in the inflammatory subset.

### Conclusions and limitations

A primary role of bioinformatics in complex diseases is to pare down the possibilities to a coherent set of candidates for future study. The many risk alleles for SSc each have modest odds ratio and the final picture of SSc will likely lie in the *interactions* between various risk factors, but the number of possible interactions between these combinatorial factors is prohibitively large. It is here that the network approach may be most useful in delineating candidates for interaction studies. We might speculate, for example, that SSc results from the presence of multiple, functionally distinct alleles, but that it does not matter what gene is mutated as long as the mutation has a particular functional outcome. The predicted interactions in the network suggest which alleles might be functionally related and which might be distinct from each other, as the alleles either cluster within a subnetwork or straddle the subnetworks.

This report is limited by our utilization of whole skin biopsies, which are complex mixtures of cells, and in that the studies were observational. The use of whole skin means that we cannot directly ascribe gene expression to specific cell types. For example, we *infer* that the M2 macrophage subnetwork is related to that cell type based on the coherent expression of monocyte markers and cytokines related to M2 polarization of macrophages. Our study is therefore *hypothesis generating*. Mechanistic studies will be needed to evaluate the existence of the molecular links suggested by the network analysis.

Nevertheless, our analyses place the intrinsic subsets as a possible readout of SSc pathology. The consensus gene expression of the subsets implicates a number of molecular mechanisms that have been associated with SSc and suggests functional roles for a large fraction of the replicated SSc-associated polymorphisms. We demonstrate that the core molecular processes of the inflammatory and fibroproliferative subsets are molecularly connected to each other. This suggests the possibility that SSc subsets may be dynamic and interconnected.

## Materials and Methods

### Ethics statement

The analysis of prospectively collected human samples in this study was approved by the Committee for the Protection of Human Subjects at Dartmouth College (CPHS#16631) and by the IRB review panel at Northwestern Feinberg School of Medicine (STU00004428). All subjects in the study provided written consent, which was approved by the IRB review panels at Dartmouth College and Northwestern Feinberg School of Medicine.

### Patient information

This study used data from three previously published cohorts (Table 1). Each of the studies is available from NCBI GEO at the following accession numbers: Milano et al. (GSE9285), Pendergrass et al. (GSE32413) and Hinchcliff et al. (GSE45485). We used an expanded version of the Hinchcliff dataset that contained an additional 12 SSc patients, 1 healthy control and 1 morphea patient beyond what was included in Hinchcliff et al.[4] (GSE59785).

Each of the three study cohorts contained patients with SSc defined using the 1980 ACR criteria. Specifically, all patients met the American College of Rheumatology classification criteria for SSc [75] and were further characterized as the diffuse (dSSc), or the limited (lSSc) subsets. Limited SSc patients had 3 of the 5 features of CREST syndrome, or had Raynaud's phenomenon with abnormal nail fold capillaries and scleroderma-specific autoantibodies. 

### Preprocessing and clustering of microarray data

All three studies used Agilent Technologies 44,000 element DNA microarrays representing the full human genome. All samples were processed and all microarrays hybridized in the Whitfield lab providing consistency between the datasets. The DNA probes between these datasets are identical and thus were indexed using the same probe identifiers allowing direct mapping from one data set to another without significant loss of data. Microarray data from each cohort were Log2 Lowess-normalized and only spots with mean fluorescent signal at least 1.5 greater than median local background in Cy3- or Cy5- channels were included in the analysis. Genes with less than 80% good data were excluded. Since a common reference experimental design was used for all cohorts, each probe was centered on its median value across all arrays. Data were multiplied by -1 to convert them to Log2(Cy3/Cy5) ratios.

The three cohorts were clustered into coexpression modules using the WGCNA procedure. We used the WGCNA R package available on the Comprehensive R Archive Network (http://cran.r-project.org) and described in [10]. We used the default parameters for running the software except that we used the “signed” network option and a soft thresholding parameter d = 12. These parameters are described in depth in [10], [15]. Genes that were classified as outliers were discarded from further analysis.

### The information graph and consensus clusters

To each pair of modules from different datasets we associate an overlap score W. Specifically, if C_i_ is a module in, say, Milano et al. and C_j_ a module in Pendergrass et al., then we define
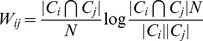
where N is the total number of genes in the genome. The W-scores can be interpreted as edge weights in a module-module network (the information graph). This network encodes the mutual information between the WGCNA-derived genomic partitions. We computed the W-scores between each pair of modules across all three datasets by the above formula and set the small and negative W-scores below a threshold to zero (S4 Fig.). A mathematical derivation of the relationship between the W-scores and mutual information and a detailed description of the thresholding procedure are available in the supporting information (S1 Text). The resulting 3-partite information graph was mined for consensus clusters.

Since triangles in the information graph represent a module conserved across all three datasets, we clustered the information graph using a variant of triangle percolation [17], which is a community detection procedure designed to find sets of modules that are members of many triangles together. Specifically, from the information graph we constructed an auxiliary graph, called the triangle graph, and detected communities in the triangle graph by greedy modularity maximization [76]. A description of the construction of the triangle graph is available in the supporting information (S1 Text).

We define a final consensus cluster as all of the genes that are contained in a module from the community for each of the three data sets community. Note that triangle percolation allows for overlapping communities in the underlying information graph. For example, the inflammatory consensus cluster and the keratinocyte consensus cluster overlap by one module (Fig. 3A). This is one of MICC's strengths because it does not require a whole module from one dataset to be associated with only one consensus cluster. To derive a gene set associated to the consensus cluster, we took all modules within that community, computed their unions within their dataset, and then computed their intersection across datasets. In symbols, let *Comm* denote a set of modules that form a community in the information graph (e.g. the dotted circles Fig. 1B and the colored nodes of Fig. 3A). Let *M_Comm_*, *P_Comm_*, and *H_Comm_* denote respectively the sets of Milano, Pendergrass and Hinchcliff modules within *Comm*. Let *m*, *p*, and *h* denote modules in the Milano, Pendergrass, and Hinchcliff data sets respectively; note that these are sets of genes. We associate a gene set *CC*_Comm_ with the community *Comm* through the following formula:
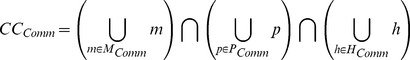
We call *CC_Comm_* the *consensus cluster associated with the community Comm* and it consists of all genes that are present in a module from each data set within the community. The elements of *CC_Comm_* are the consensus genes. It is clear by definition that the consensus clusters are nonoverlapping even though communities can share modules. This is because a gene needs to be present in a module in the community from each of the three data sets. Since modules do not overlap within data sets, consensus clusters cannot either.

### Statistical tests for subset specificity

To determine if a WGCNA-derived module was significantly differentially regulated in a subset, we performed one-tailed Wilcoxon rank sum tests. Specifically, we computed the module eigengene of each module by first normalizing the gene expression so that each gene expression vector had Euclidean length 1. The module eigengene is the first principal component of the normalized gene expression vectors within the WGCNA module. The module eigengene is a one-dimensional summary score for the module's gene expression across all biopsies. To determine if the module was significantly up- or down-regulated in a particular subset, we determined if the median of the module eigengene for that subset was above or below that of the whole population, and then performed a one-tailed Wilcoxon rank sum test to determine the significance of the median being above or below that of the population as a whole. We used the subset assignments reported in the previous papers describing these datasets [1], [4], [11]. We used Bonferroni corrections for multiple comparisons. There were 178 modules in total across the datasets. In Table 2, we corrected for 178×3 tests for each of the subset-specificity tests. In Fig. 3B, we corrected for 178×4 tests because we included tests for all non-normal-like SSc versus normal-like SSc and healthy controls (see also S4 Data file).

### The IMP Bayesian network

The IMP Bayesian network is available through an online interface at (http://imp.princeton.edu). To build our network, we queried IMP with four gene sets: inflammatory and fibroproliferative consensus genes derived from the consensus clusters, SSc-associated polymorphisms (as described below), and the four gene MRSS biomarker reported in [5]. IMP provides export of the subnetwork corresponding to the query genes as a weighted edge list (a three-column table indicating which genes are connected and with what probability). IMP automatically thresholds the probabilities at 0.5 and exports the network with up to an additional 50 genes that provide extra context for the query genes. In our case, the 50 genes were predominantly cell cycle genes. This is probably because the cell cycle is heavily studied in the microarray compendium from which IMP was built. In that case, IMP would be highly confident about predicting interactions between the fibroproliferative genes and other cell cycle genes.

We developed in-house Matlab and R scripts to transform the edge list data into the Graph Exchange Format (gexf), which allows for manipulation in Gephi, an open source network visualization program [77]. Data files S7-S8 contain post-processed networks and Data files S10-S11 provide R data and code snippets for manipulating the network programmatically.

### Genetic polymorphisms for network analysis

We collected genes with SSc-associated polymorphisms from the literature and curated them according to the following criteria. We included polymorphic genes that were reported in genome-wide association studies of SSc [78]–[82], from a recent study using the Immunochip platform [83] and from case-control candidate gene studies that were replicated in at least one other study [54], [84]–[105]. This resulted in a list of 41 polymorphic genes (S6 Data file).

## Supporting Information

S1 Fig Consensus genes are enriched for coexpression hubs. The consensus genes reported by MICC are more correlated to their module eigengene (red density) than is typical for an arbitrary gene-eigengene correlation (blue density). Genes are compared only to their module eigengene, i.e. to the hub that the gene is closest to in the coexpression network. Note the evident shift in the red density toward 1, which is perfect correlation, indicating that consensus genes are more “hub-like”.
(TIF)

S2 Fig Adjacency matrix for the triangle graph. The triangle graph is a weighted graph whose nodes are *triangles* in the information graph and whose edges indicate that the corresponding triangles in the information graph *share and edge*. (**A**) The weighted adjacency matrix for the triangle graph. Rows and columns of the adjacency matrix are indexed by nodes of the triangle graph (i.e. by triangles in the information graph). The rows and columns of the matrix are sorted according to community order. Note the distinct block structure of the matrix indicating that the underlying graph is highly modular. (**B**) The same matrix, but *unweighted* so that the matrix contains only 0's and 1's (blue and red cells in the matrix, respectively) indicating that the nodes are either connected or disconnected. This aids in the visualization of the community structure of the graph (block structure of the matrix), although community detection was performed on the weighted triangle graph.
(TIF)

S3 Fig Schematic for building consensus gene sets. To each community (1) in the information graph we associate a consensus gene set by (2) computing the union of modules *within* a data set and then (3) computing the intersection across data sets.
(TIF)

S4 Fig Construction of the information graph. (**A**) Three pairs of partitions of a 12-element set and their associated bipartite information graphs. Edge width denotes the size of the W-score for a pair of modules. Dotted edges represent *negative* W-scores. The highest possible mutual information occurs when modules are perfectly conserved. The information graph is disconnected with edges denoting the mapping between conserved modules. In the intermediate case, modules break into pieces that are reassorted among each other. The information graph here has strong community structure, but is not completely disconnected. The low mutual information case occurs when the partitions labels are random with respect to each other. In this case, all edges are small and are partially cancelled by the negative edges also present in the graph. (**B**,**C**) W-scores are calculated for each pair of modules; in this case one from Milano and one from Pendergrass. (**B**) Most W-scores are small in absolute value (blue histogram; logarithm of density), while their distribution has a right tail of significantly large scores. We can threshold the small and negative W-scores by keeping only those scores that contribute positively to the total mutual information (red curve; x-intercept). The sum of all W-scores is the total mutual information between the Milano and Pendergrass genomic partitions (dashed blue horizontal line). (**C**) The W-scores are positively correlated with the size of the overlap between gene clusters, but the relationship is not perfect. The W-score threshold is shown by a dotted blue vertical line and the overlaps that exceed the threshold are plotted in red. In particular, note that there are relatively large overlaps that fail to meet the threshold. Likewise, there are relatively small overlaps that have high W-scores.
(TIF)

S1 Text Additional mathematical details about the MICC method and glossary of keywords used in main text.
(PDF)

S1 Data file WGCNA clustered PCL file for Milano skin data.
(ZIP)

S2 Data file WGCNA clustered PCL file for Pendergrass skin data.
(ZIP)

S3 Data file WGCNA clustered PCL file for Hinchcliff skin data.
(ZIP)

S4 Data file Table of p-values for modules in each dataset (includes module sizes).
(XLSX)

S5 Data file Full output of g:Profiler for consensus clusters.
(XLS)

S6 Data file Complete list of polymorphic genes used in this study.
(XLSX)

S7 Data file Molecular network plotting file GEXF format.
(GEXF)

S8 Data file Molecular network plotting file Gephi format.
(ZIP)

S9 Data file Molecular network plotted in PDF (text searchable for genes).
(PDF)

S10 Data file R data for programmatic access to network (iGraph format).
(ZIP)

S11 Data file R code snippet demonstrating reading and writing graphs from R to GEXF format.
(R) Click here for additional data file.

## References

[1] Milano A, Pendergrass SA, Sargent JL, George LK, McCalmont TH, et al (2008) Molecular subsets in the gene expression signatures of scleroderma skin. PLoS ONE 3: e2696. 18648520 10.1371/journal.pone.0002696 PMC2481301

[2] Whitfield ML, George LK, Grant GD, Perou CM (2006) Common markers of proliferation. Nat Rev Cancer 6: 99–106. 16491069 10.1038/nrc1802

[3] Steen VD, Medsger TA (2007) Changes in causes of death in systemic sclerosis, 1972–2002. Ann Rheum Dis 66: 940–944. 17329309 10.1136/ard.2006.066068 PMC1955114

[4] Hinchcliff M, Huang C-C, Wood TA, Matthew Mahoney J, Martyanov V, et al. (2013) Molecular Signatures in Skin Associated with Clinical Improvement during Mycophenolate Treatment in Systemic Sclerosis. J Invest Dermatol: -. 10.1038/jid.2013.130 PMC3714324 23677167

[5] Farina G, Lafyatis D, Lemaire R, Lafyatis R (2010) A four-gene biomarker predicts skin disease in patients with diffuse cutaneous systemic sclerosis. Arthritis Rheum 62: 580–588. 20112379 10.1002/art.27220 PMC3018285

[6] Eisen MB, Spellman PT, Brown PO, Botstein D (1998) Cluster analysis and display of genome-wide expression patterns. Proc Natl Acad Sci U S A 95: 14863–14868. 9843981 10.1073/pnas.95.25.14863 PMC24541

[7] Whitfield ML, Sherlock G, Saldanha AJ, Murray JI, Ball CA, et al (2002) Identification of genes periodically expressed in the human cell cycle and their expression in tumors. Mol Biol Cell 13: 1977–2000. 12058064 10.1091/mbc.02-02-0030. PMC117619

[8] Barabasi AL (2007) Network medicine—from obesity to the “diseasome”. N Engl J Med 357: 404–407. 17652657 10.1056/NEJMe078114

[9] Barabasi AL, Gulbahce N, Loscalzo J (2011) Network medicine: a network-based approach to human disease. Nat Rev Genet 12: 56–68. 21164525 10.1038/nrg2918 PMC3140052

[10] Langfelder P, Horvath S (2008) WGCNA: an R package for weighted correlation network analysis. BMC Bioinformatics 9: 559. 19114008 10.1186/1471-2105-9-559 PMC2631488

[11] Pendergrass SA, Lemaire R, Francis IP, Mahoney JM, Lafyatis R, et al (2012) Intrinsic gene expression subsets of diffuse cutaneous systemic sclerosis are stable in serial skin biopsies. J Invest Dermatol 132: 1363–1373. 22318389 10.1038/jid.2011.472 PMC3326181

[12] Strehl A, Ghosh J (2003) Cluster ensembles—a knowledge reuse framework for combining multiple partitions. The Journal of Machine Learning Research 3: 583–617.

[13] Monti ST, Pablo; Mesirov, Jill; Golub, Todd (2003) Consensus clustering: a resampling-based method for class discovery and visualization of gene expression microarray data. Machine learning 52.

[14] Cover TM, Thomas JA (2012) Elements of information theory: Wiley-interscience.

[15] Horvath S, Dong J (2008) Geometric interpretation of gene coexpression network analysis. PLoS Comput Biol 4: e1000117. 18704157 10.1371/journal.pcbi.1000117 PMC2446438

[16] Whitfield ML, Finlay DR, Murray JI, Troyanskaya OG, Chi JT, et al (2003) Systemic and cell type-specific gene expression patterns in scleroderma skin. Proc Natl Acad Sci U S A 100: 12319–12324. 14530402 10.1073/pnas.1635114100 PMC218756

[17] Palla G, Derenyi I, Farkas I, Vicsek T (2005) Uncovering the overlapping community structure of complex networks in nature and society. Nature 435: 814–818. 15944704 10.1038/nature03607

[18] Reimand J, Arak T, Vilo J (2011) g:Profiler-a web server for functional interpretation of gene lists (2011 update). Nucleic Acids Res 39: W307–315. 21646343 10.1093/nar/gkr378 PMC3125778

[19] Johnson ME, Mahoney JM, Taroni J, Sargent JL, Marmarelis E, et al. (in press) Experimentally-derived fibroblast gene signatures identify molecular pathways associated with distinct subsets of systemic sclerosis patients in three independent cohorts. PLoS ONE, 10.1371/journal.pone.0114017. 10.1371/journal.pone.0114017 PMC4301872 25607805

[20] Wong AK, Park CY, Greene CS, Bongo LA, Guan Y, et al (2012) IMP: a multi-species functional genomics portal for integration, visualization and prediction of protein functions and networks. Nucleic Acids Res 40: W484–490. 22684505 10.1093/nar/gks458 PMC3394282

[21] Scala E, Pallotta S, Frezzolini A, Abeni D, Barbieri C, et al (2004) Cytokine and chemokine levels in systemic sclerosis: relationship with cutaneous and internal organ involvement. Clin Exp Immunol 138: 540–546. 15544634 10.1111/j.1365-2249.2004.02642.x PMC1809238

[22] Riccieri V, Rinaldi T, Spadaro A, Scrivo R, Ceccarelli F, et al (2003) Interleukin-13 in systemic sclerosis: relationship to nailfold capillaroscopy abnormalities. Clin Rheumatol 22: 102–106. 12740673 10.1007/s10067-002-0684-z

[23] Auffray C, Fogg D, Garfa M, Elain G, Join-Lambert O, et al (2007) Monitoring of blood vessels and tissues by a population of monocytes with patrolling behavior. Science 317: 666–670. 17673663 10.1126/science.1142883

[24] Katakura T, Miyazaki M, Kobayashi M, Herndon DN, Suzuki F (2004) CCL17 and IL-10 as effectors that enable alternatively activated macrophages to inhibit the generation of classically activated macrophages. J Immunol 172: 1407–1413. 14734716 10.4049/jimmunol.172.3.1407

[25] Weis N, Weigert A, von Knethen A, Brune B (2009) Heme oxygenase-1 contributes to an alternative macrophage activation profile induced by apoptotic cell supernatants. Mol Biol Cell 20: 1280–1288. 19129475 10.1091/mbc.E08-10-1005 PMC2649271

[26] Higashi-Kuwata N, Makino T, Inoue Y, Takeya M, Ihn H (2009) Alternatively activated macrophages (M2 macrophages) in the skin of patient with localized scleroderma. Exp Dermatol 18: 727–729. 19320738 10.1111/j.1600-0625.2008.00828.x

[27] Gordon S, Martinez FO (2010) Alternative activation of macrophages: mechanism and functions. Immunity 32: 593–604. 20510870 10.1016/j.immuni.2010.05.007

[28] Mosser DM, Edwards JP (2008) Exploring the full spectrum of macrophage activation. Nat Rev Immunol 8: 958–969. 19029990 10.1038/nri2448 PMC2724991

[29] Atamas SP, White B (2003) Cytokine regulation of pulmonary fibrosis in scleroderma. Cytokine Growth Factor Rev 14: 537–550. 14563355 10.1016/s1359-6101(03)00060-1

[30] Zhou W, Zhang F, Aune TM (2003) Either IL-2 or IL-12 is sufficient to direct Th1 differentiation by nonobese diabetic T cells. J Immunol 170: 735–740. 12517935 10.4049/jimmunol.170.2.735

[31] Gollob JA, Murphy EA, Mahajan S, Schnipper CP, Ritz J, et al (1998) Altered interleukin-12 responsiveness in Th1 and Th2 cells is associated with the differential activation of STAT5 and STAT1. Blood 91: 1341–1354. 9454765

[32] Naeger LK, McKinney J, Salvekar A, Hoey T (1999) Identification of a STAT4 binding site in the interleukin-12 receptor required for signaling. J Biol Chem 274: 1875–1878. 9890938 10.1074/jbc.274.4.1875

[33] O'Shea JJ, Lahesmaa R, Vahedi G, Laurence A, Kanno Y (2011) Genomic views of STAT function in CD4+ T helper cell differentiation. Nat Rev Immunol 11: 239–250. 21436836 10.1038/nri2958 PMC3070307

[34] Mackay IR (2009) Clustering and commonalities among autoimmune diseases. J Autoimmun 33: 170–177. 19837564 10.1016/j.jaut.2009.09.006

[35] de Paus RA, Geilenkirchen MA, van Riet S, van Dissel JT, van de Vosse E (2013) Differential expression and function of human IL-12Rbeta2 polymorphic variants. Mol Immunol 56: 380–389. 23911393 10.1016/j.molimm.2013.07.002

[36] Yelo E, Bernardo MV, Gimeno L, Alcaraz-Garcia MJ, Majado MJ, et al (2008) Dock10, a novel CZH protein selectively induced by interleukin-4 in human B lymphocytes. Mol Immunol 45: 3411–3418. 18499258 10.1016/j.molimm.2008.04.003

[37] Xu Y, Harder KW, Huntington ND, Hibbs ML, Tarlinton DM (2005) Lyn tyrosine kinase: accentuating the positive and the negative. Immunity 22: 9–18. 15664155 10.1016/j.immuni.2004.12.004

[38] Silver K, Bouriez-Jones T, Crockford T, Ferry H, Tang HL, et al (2006) Spontaneous class switching and B cell hyperactivity increase autoimmunity against intracellular self antigen in Lyn-deficient mice. Eur J Immunol 36: 2920–2927. 17039569 10.1002/eji.200636462

[39] Hata A, Sabe H, Kurosaki T, Takata M, Hanafusa H (1994) Functional analysis of Csk in signal transduction through the B-cell antigen receptor. Mol Cell Biol 14: 7306–7313. 7935444 10.1128/mcb.14.11.7306-7313.1994 PMC359265

[40] Manjarrez-Orduno N, Marasco E, Chung SA, Katz MS, Kiridly JF, et al (2012) CSK regulatory polymorphism is associated with systemic lupus erythematosus and influences B-cell signaling and activation. Nat Genet 44: 1227–1230. 23042117 10.1038/ng.2439 PMC3715052

[41] Levinson NM, Seeliger MA, Cole PA, Kuriyan J (2008) Structural basis for the recognition of c-Src by its inactivator Csk. Cell 134: 124–134. 18614016 10.1016/j.cell.2008.05.051 PMC2494536

[42] Vang T, Liu WH, Delacroix L, Wu S, Vasile S, et al (2012) LYP inhibits T-cell activation when dissociated from CSK. Nat Chem Biol 8: 437–446. 22426112 10.1038/nchembio.916 PMC3329573

[43] Fiorillo E, Orru V, Stanford SM, Liu Y, Salek M, et al (2010) Autoimmune-associated PTPN22 R620W variation reduces phosphorylation of lymphoid phosphatase on an inhibitory tyrosine residue. J Biol Chem 285: 26506–26518. 20538612 10.1074/jbc.M110.111104 PMC2924087

[44] Burn GL, Svensson L, Sanchez-Blanco C, Saini M, Cope AP (2011) Why is PTPN22 a good candidate susceptibility gene for autoimmune disease? FEBS Lett 585: 3689–3698. 21515266 10.1016/j.febslet.2011.04.032

[45] Yamamoto K, Yamaguchi M, Miyasaka N, Miura O (2003) SOCS-3 inhibits IL-12-induced STAT4 activation by binding through its SH2 domain to the STAT4 docking site in the IL-12 receptor beta2 subunit. Biochem Biophys Res Commun 310: 1188–1193. 14559241 10.1016/j.bbrc.2003.09.140

[46] Varga J, Whitfield ML (2009) Transforming growth factor-beta in systemic sclerosis (scleroderma). Front Biosci (Schol Ed) 1: 226–235. 19482698 10.2741/s22

[47] Katsumoto TR, Whitfield ML, Connolly MK (2011) The pathogenesis of systemic sclerosis. Annu Rev Pathol 6: 509–537. 21090968 10.1146/annurev-pathol-011110-130312

[48] Perou CM, Sorlie T, Eisen MB, van de Rijn M, Jeffrey SS, et al (2000) Molecular portraits of human breast tumours. Nature 406: 747–752. 10963602 10.1038/35021093

[49] Liu H, Thaker YR, Stagg L, Schneider H, Ladbury JE, et al (2013) SLP-76 sterile alpha motif (SAM) and individual H5 alpha helix mediate oligomer formation for microclusters and T-cell activation. J Biol Chem 288: 29539–29549. 23935094 10.1074/jbc.M112.424846 PMC3795252

[50] Rege TA, Hagood JS (2006) Thy-1, a versatile modulator of signaling affecting cellular adhesion, proliferation, survival, and cytokine/growth factor responses. Biochim Biophys Acta 1763: 991–999. 16996153 10.1016/j.bbamcr.2006.08.008 PMC1781924

[51] Cohen PY, Breuer R, Wallach-Dayan SB (2009) Thy1 up-regulates FasL expression in lung myofibroblasts via Src family kinases. Am J Respir Cell Mol Biol 40: 231–238. 18676775 10.1165/rcmb.2007-0348OC

[52] Phipps RP, Penney DP, Keng P, Quill H, Paxhia A, et al (1989) Characterization of two major populations of lung fibroblasts: distinguishing morphology and discordant display of Thy 1 and class II MHC. Am J Respir Cell Mol Biol 1: 65–74. 2576218 10.1165/ajrcmb/1.1.65

[53] Yamamoto A, Ashihara E, Nakagawa Y, Obayashi H, Ohta M, et al (2011) Allograft inflammatory factor-1 is overexpressed and induces fibroblast chemotaxis in the skin of sclerodermatous GVHD in a murine model. Immunol Lett 135: 144–150. 21040744 10.1016/j.imlet.2010.10.015

[54] Manetti M, Allanore Y, Revillod L, Fatini C, Guiducci S, et al (2011) A genetic variation located in the promoter region of the UPAR (CD87) gene is associated with the vascular complications of systemic sclerosis. Arthritis Rheum 63: 247–256. 20967855 10.1002/art.30101

[55] Saris CG, Horvath S, van Vught PW, van Es MA, Blauw HM, et al (2009) Weighted gene co-expression network analysis of the peripheral blood from Amyotrophic Lateral Sclerosis patients. BMC Genomics 10: 405. 19712483 10.1186/1471-2164-10-405 PMC2743717

[56] Farber CR, Bennett BJ, Orozco L, Zou W, Lira A, et al (2011) Mouse genome-wide association and systems genetics identify Asxl2 as a regulator of bone mineral density and osteoclastogenesis. PLoS Genet 7: e1002038. 21490954 10.1371/journal.pgen.1002038 PMC3072371

[57] Horvath S, Nazmul-Hossain AN, Pollard RP, Kroese FG, Vissink A, et al (2012) Systems analysis of primary Sjogren's syndrome pathogenesis in salivary glands identifies shared pathways in human and a mouse model. Arthritis Res Ther 14: R238. 23116360 10.1186/ar4081 PMC3674589

[58] Lemaire R, Farina G, Kissin E, Shipley JM, Bona C, et al (2004) Mutant fibrillin 1 from tight skin mice increases extracellular matrix incorporation of microfibril-associated glycoprotein 2 and type I collagen. Arthritis Rheum 50: 915–926. 15022335 10.1002/art.20053

[59] Gerber EE, Gallo EM, Fontana SC, Davis EC, Wigley FM, et al. (2013) Integrin-modulating therapy prevents fibrosis and autoimmunity in mouse models of scleroderma. Nature. 10.1038/nature12614 PMC3992987 24107997

[60] Tan FK, Wang N, Kuwana M, Chakraborty R, Bona CA, et al (2001) Association of fibrillin 1 single-nucleotide polymorphism haplotypes with systemic sclerosis in Choctaw and Japanese populations. Arthritis Rheum 44: 893–901. 11315929 10.1002/1529-0131(200104)44:4<893::AID-ANR146>3.0.CO;2-3

[61] Tan FK, Tercero GM, Arnett FC, Wang N, Chakraborty R (2003) Examination of the possible role of biologically relevant genes around FBN1 in systemic sclerosis in the Choctaw population. Arthritis Rheum 48: 3295–3296. 14613297 10.1002/art.11280

[62] Zhou X, Tan FK, Wang N, Xiong M, Maghidman S, et al (2003) Genome-wide association study for regions of systemic sclerosis susceptibility in a Choctaw Indian population with high disease prevalence. Arthritis Rheum 48: 2585–2592. 13130478 10.1002/art.11220

[63] Alkassab F, Gourh P, Tan FK, McNearney T, Fischbach M, et al (2007) An allograft inflammatory factor 1 (AIF1) single nucleotide polymorphism (SNP) is associated with anticentromere antibody positive systemic sclerosis. Rheumatology (Oxford) 46: 1248–1251. 17522098 10.1093/rheumatology/kem057

[64] Sibinga NE, Feinberg MW, Yang H, Werner F, Jain MK (2002) Macrophage-restricted and interferon gamma-inducible expression of the allograft inflammatory factor-1 gene requires Pu.1. J Biol Chem 277: 16202–16210. 11861656 10.1074/jbc.M200935200

[65] Tian Y, Jain S, Kelemen SE, Autieri MV (2009) AIF-1 expression regulates endothelial cell activation, signal transduction, and vasculogenesis. Am J Physiol, Cell Physiol 296: C256–266. 18787073 10.1152/ajpcell.00325.2008 PMC2643850

[66] Greenblatt MB, Sargent JL, Farina G, Tsang K, Lafyatis R, et al (2012) Interspecies comparison of human and murine scleroderma reveals IL-13 and CCL2 as disease subset-specific targets. Am J Pathol 180: 1080–1094. 22245215 10.1016/j.ajpath.2011.11.024 PMC3349888

[67] Mantovani A, Garlanda C, Locati M (2009) Macrophage diversity and polarization in atherosclerosis: a question of balance. Arterioscler Thromb Vasc Biol 29: 1419–1423. 19696407 10.1161/ATVBAHA.108.180497

[68] Mantovani A, Sica A, Sozzani S, Allavena P, Vecchi A, et al (2004) The chemokine system in diverse forms of macrophage activation and polarization. Trends Immunol 25: 677–686. 15530839 10.1016/j.it.2004.09.015

[69] Arai M, Ikawa Y, Chujo S, Hamaguchi Y, Ishida W, et al (2013) Chemokine receptors CCR2 and CX3CR1 regulate skin fibrosis in the mouse model of cytokine-induced systemic sclerosis. J Dermatol Sci 69: 250–258. 23142052 10.1016/j.jdermsci.2012.10.010

[70] Roca H, Varsos ZS, Sud S, Craig MJ, Ying C, et al (2009) CCL2 and interleukin-6 promote survival of human CD11b+ peripheral blood mononuclear cells and induce M2-type macrophage polarization. J Biol Chem 284: 34342–34354. 19833726 10.1074/jbc.M109.042671 PMC2797202

[71] Saito E, Fujimoto M, Hasegawa M, Komura K, Hamaguchi Y, et al (2002) CD19-dependent B lymphocyte signaling thresholds influence skin fibrosis and autoimmunity in the tight-skin mouse. J Clin Invest 109: 1453–1462. 12045259 10.1172/JCI15078 PMC150999

[72] Opitz C, Klein-Weigel PF, Riemekasten G (2011) Systemic sclerosis - a systematic overview: part 2 - immunosuppression, treatment of SSc-associated vasculopathy, and treatment of pulmonary arterial hypertension. VASA 40: 20–30. 21283970 10.1024/0301-1526/a000066

[73] Arron ST, Dimon MT, Li Z, Johnson ME, T AW, et al (2014) High Rhodotorula sequences in skin transcriptome of patients with diffuse systemic sclerosis. J Invest Dermatol 134: 2138–2145. 24608988 10.1038/jid.2014.127 PMC4102619

[74] Farina A, Cirone M, York M, Lenna S, Padilla C, et al (2014) Epstein-Barr virus infection induces aberrant TLR activation pathway and fibroblast-myofibroblast conversion in scleroderma. J Invest Dermatol 134: 954–964. 24129067 10.1038/jid.2013.423 PMC3961515

[75] Committee. SfSCotARADaTC (1980) Preliminary criteria for the classification of systemic sclerosis (scleroderma). 23: 581–590.

[76] Newman MEJ (2006) Modularity and community structure in networks. Proceedings of the National Academy of Sciences 103: 8577–8582. 10.1073/pnas.0601602103 PMC1482622 16723398

[77] Bastian M, Heymann S, Jacomy M. Gephi: an open source software for exploring and manipulating networks.; 2009.

[78] Radstake TR, Gorlova O, Rueda B, Martin JE, Alizadeh BZ, et al (2010) Genome-wide association study of systemic sclerosis identifies CD247 as a new susceptibility locus. Nat Genet 42: 426–429. 20383147 10.1038/ng.565 PMC2861917

[79] Gorlova O, Martin JE, Rueda B, Koeleman BP, Ying J, et al (2011) Identification of novel genetic markers associated with clinical phenotypes of systemic sclerosis through a genome-wide association strategy. PLoS Genet 7: e1002178. 21779181 10.1371/journal.pgen.1002178 PMC3136437

[80] Allanore Y, Saad M, Dieude P, Avouac J, Distler JH, et al (2011) Genome-wide scan identifies TNIP1, PSORS1C1, and RHOB as novel risk loci for systemic sclerosis. PLoS Genet 7: e1002091. 21750679 10.1371/journal.pgen.1002091 PMC3131285

[81] Martin JE, Broen JC, Carmona FD, Teruel M, Simeon CP, et al (2012) Identification of CSK as a systemic sclerosis genetic risk factor through Genome Wide Association Study follow-up. Hum Mol Genet 21: 2825–2835. 22407130 10.1093/hmg/dds099 PMC3368627

[82] Martin JE, Assassi S, Diaz-Gallo LM, Broen JC, Simeon CP, et al (2013) A systemic sclerosis and systemic lupus erythematosus pan-meta-GWAS reveals new shared susceptibility loci. Hum Mol Genet 22: 4021–4029. 23740937 10.1093/hmg/ddt248 PMC3766185

[83] Mayes MD, Bossini-Castillo L, Gorlova O, Martin JE, Zhou X, et al (2014) Immunochip analysis identifies multiple susceptibility loci for systemic sclerosis. Am J Hum Genet 94: 47–61. 24387989 10.1016/j.ajhg.2013.12.002 PMC3882906

[84] Rueda B, Gourh P, Broen J, Agarwal SK, Simeon C, et al (2010) BANK1 functional variants are associated with susceptibility to diffuse systemic sclerosis in Caucasians. Ann Rheum Dis 69: 700–705. 19815934 10.1136/ard.2009.118174 PMC2975737

[85] Gourh P, Agarwal SK, Martin E, Divecha D, Rueda B, et al (2010) Association of the C8orf13-BLK region with systemic sclerosis in North-American and European populations. J Autoimmun 34: 155–162. 19796918 10.1016/j.jaut.2009.08.014 PMC2821978

[86] Manetti M, Allanore Y, Saad M, Fatini C, Cohignac V, et al (2012) Evidence for caveolin-1 as a new susceptibility gene regulating tissue fibrosis in systemic sclerosis. Ann Rheum Dis 71: 1034–1041. 22402147 10.1136/annrheumdis-2011-200986

[87] Koumakis E, Bouaziz M, Dieude P, Ruiz B, Riemekasten G, et al (2013) A regulatory variant in CCR6 is associated with susceptibility to antitopoisomerase-positive systemic sclerosis. Arthritis Rheum 65: 3202–3208. 23983073 10.1002/art.38136

[88] Dieude P, Guedj M, Truchetet ME, Wipff J, Revillod L, et al (2011) Association of the CD226 Ser(307) variant with systemic sclerosis: evidence of a contribution of costimulation pathways in systemic sclerosis pathogenesis. Arthritis Rheum 63: 1097–1105. 21162102 10.1002/art.30204

[89] Hoshino K, Satoh T, Kawaguchi Y, Kuwana M (2011) Association of hepatocyte growth factor promoter polymorphism with severity of interstitial lung disease in Japanese patients with systemic sclerosis. Arthritis Rheum 63: 2465–2472. 21520010 10.1002/art.30415

[90] Diaz-Gallo LM, Simeon CP, Broen JC, Ortego-Centeno N, Beretta L, et al (2013) Implication of IL-2/IL-21 region in systemic sclerosis genetic susceptibility. Ann Rheum Dis 72: 1233–1238. 23172754 10.1136/annrheumdis-2012-202357 PMC3887514

[91] Bossini-Castillo L, Martin JE, Broen J, Gorlova O, Simeon CP, et al (2012) A GWAS follow-up study reveals the association of the IL12RB2 gene with systemic sclerosis in Caucasian populations. Hum Mol Genet 21: 926–933. 22076442 10.1093/hmg/ddr522 PMC3298110

[92] Dieude P, Bouaziz M, Guedj M, Riemekasten G, Airo P, et al (2011) Evidence of the contribution of the X chromosome to systemic sclerosis susceptibility: association with the functional IRAK1 196Phe/532Ser haplotype. Arthritis Rheum 63: 3979–3987. 21898345 10.1002/art.30640

[93] Dieude P, Guedj M, Wipff J, Avouac J, Fajardy I, et al (2009) Association between the IRF5 rs2004640 functional polymorphism and systemic sclerosis: a new perspective for pulmonary fibrosis. Arthritis Rheum 60: 225–233. 19116937 10.1002/art.24183

[94] Sharif R, Mayes MD, Tan FK, Gorlova OY, Hummers LK, et al (2012) IRF5 polymorphism predicts prognosis in patients with systemic sclerosis. Ann Rheum Dis 71: 1197–1202. 22440820 10.1136/annrheumdis-2011-200901 PMC3375372

[95] Dieude P, Dawidowicz K, Guedj M, Legrain Y, Wipff J, et al (2010) Phenotype-haplotype correlation of IRF5 in systemic sclerosis: role of 2 haplotypes in disease severity. J Rheumatol 37: 987–992. 20231204 10.3899/jrheum.091163

[96] Carmona FD, Gutala R, Simeon CP, Carreira P, Ortego-Centeno N, et al (2012) Novel identification of the IRF7 region as an anticentromere autoantibody propensity locus in systemic sclerosis. Ann Rheum Dis 71: 114–119. 21926187 10.1136/annrheumdis-2011-200275 PMC3369428

[97] Carmona FD, Simeon CP, Beretta L, Carreira P, Vonk MC, et al (2011) Association of a non-synonymous functional variant of the ITGAM gene with systemic sclerosis. Ann Rheum Dis 70: 2050–2052. 21571730 10.1136/ard.2010.148874

[98] Anaya JM, Kim-Howard X, Prahalad S, Chernavsky A, Canas C, et al (2012) Evaluation of genetic association between an ITGAM non-synonymous SNP (rs1143679) and multiple autoimmune diseases. Autoimmun Rev 11: 276–280. 21840425 10.1016/j.autrev.2011.07.007 PMC3224188

[99] Coustet B, Agarwal SK, Gourh P, Guedj M, Mayes MD, et al (2011) Association study of ITGAM, ITGAX, and CD58 autoimmune risk loci in systemic sclerosis: results from 2 large European Caucasian cohorts. J Rheumatol 38: 1033–1038. 21362770 10.3899/jrheum.101053 PMC3404507

[100] Wu SP, Leng L, Feng Z, Liu N, Zhao H, et al (2006) Macrophage migration inhibitory factor promoter polymorphisms and the clinical expression of scleroderma. Arthritis Rheum 54: 3661–3669. 17075815 10.1002/art.22179

[101] Diaz-Gallo LM, Gourh P, Broen J, Simeon C, Fonollosa V, et al (2011) Analysis of the influence of PTPN22 gene polymorphisms in systemic sclerosis. Ann Rheum Dis 70: 454–462. 21131644 10.1136/ard.2010.130138 PMC3170726

[102] Rueda B, Broen J, Simeon C, Hesselstrand R, Diaz B, et al (2009) The STAT4 gene influences the genetic predisposition to systemic sclerosis phenotype. Hum Mol Genet 18: 2071–2077. 19286670 10.1093/hmg/ddp119

[103] Broen JC, Bossini-Castillo L, van Bon L, Vonk MC, Knaapen H, et al (2012) A rare polymorphism in the gene for Toll-like receptor 2 is associated with systemic sclerosis phenotype and increases the production of inflammatory mediators. Arthritis Rheum 64: 264–271. 21905008 10.1002/art.33325

[104] Dieude P, Guedj M, Wipff J, Ruiz B, Riemekasten G, et al (2010) Association of the TNFAIP3 rs5029939 variant with systemic sclerosis in the European Caucasian population. Ann Rheum Dis 69: 1958–1964. 20511617 10.1136/ard.2009.127928

[105] Gourh P, Arnett FC, Tan FK, Assassi S, Divecha D, et al (2010) Association of TNFSF4 (OX40L) polymorphisms with susceptibility to systemic sclerosis. Ann Rheum Dis 69: 550–555. 19778912 10.1136/ard.2009.116434 PMC2927683

