# Correction: Systems Level Analysis of Systemic Sclerosis Shows a Network of Immune and Profibrotic Pathways Connected with Genetic Polymorphisms

**DOI:** 10.1371/journal.pcbi.1004133

**Published:** 2015-03-05

**Authors:** The PLOS Computational Biology Staff

An error was introduced during the production process: in the author affiliations list of this manuscript, the place name “Hannover” should be spelt “Hanover”.

The funding statement of this manuscript should include the sentence: “MEH was supported by Award Number K23AR059763 from the National Institute of Arthritis, Musculoskeletal, and Skin Diseases (NIAMS).”
