# Supplementary figures and images for: Systems Level Analysis of Systemic Sclerosis Shows a Network of Immune and Profibrotic Pathways Connected with Genetic Polymorphisms
Source: PLoS Comput Biol. 2015 Jan 8;11(1):e1004005. doi: 10.1371/journal.pcbi.1004005 (PMC4288710; doi:10.1371/journal.pcbi.1004005)

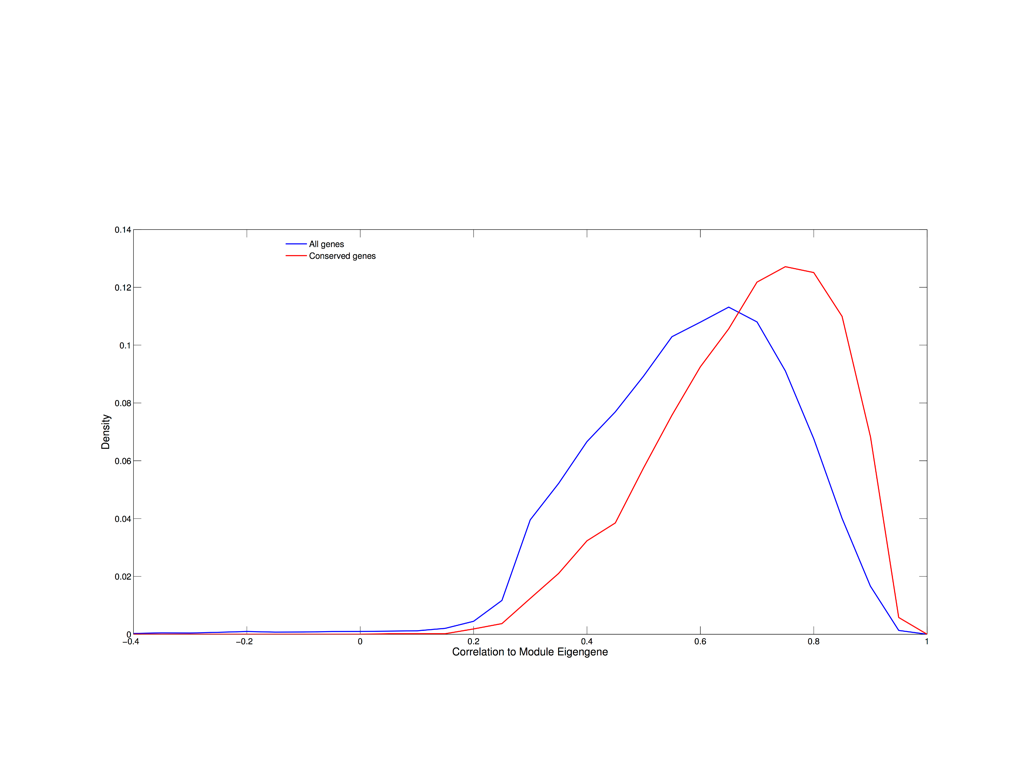

Supplement: S1 Fig — Consensus genes are enriched for coexpression hubs. The consensus genes reported by MICC are more correlated to their module eigengene (red density) than is typical for an arbitrary gene-eigengene correlation (blue density). Genes are compared only to their module eigengene, i.e. to the hub that the gene is closest to in the coexpression network. Note the evident shift in the red density toward 1, which is perfect correlation, indicating that consensus genes are more “hub-like”. (TIF) [file pcbi.1004005.s001.tif]

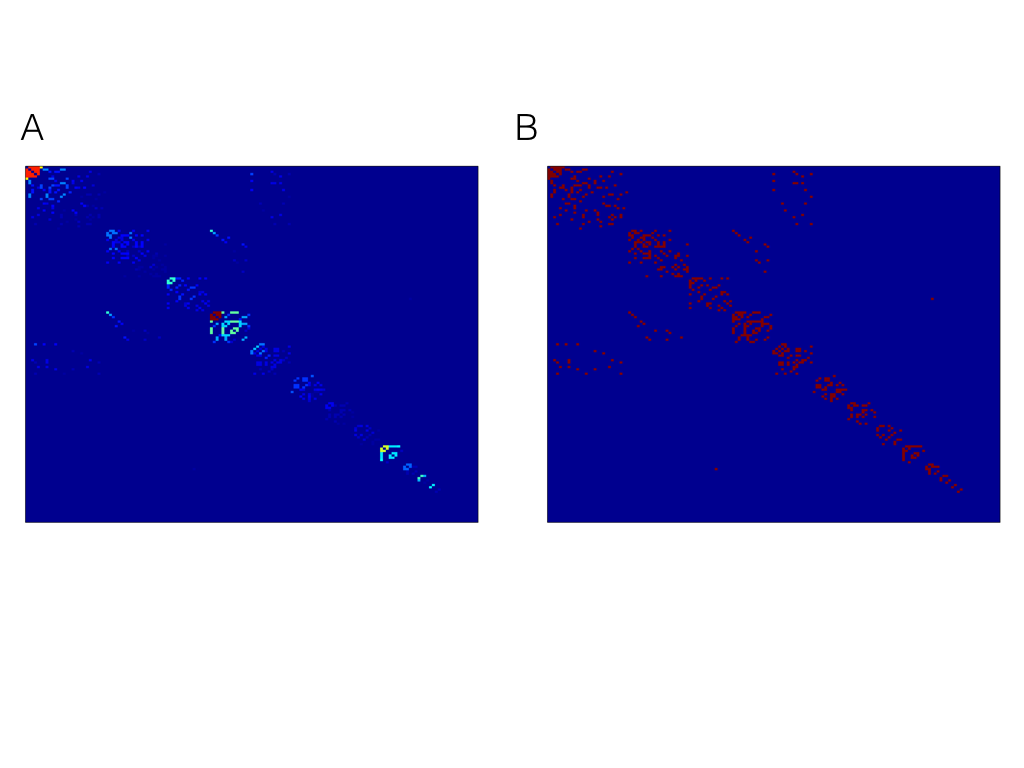

Supplement: S2 Fig — Adjacency matrix for the triangle graph. The triangle graph is a weighted graph whose nodes are triangles in the information graph and whose edges indicate that the corresponding triangles in the information graph share and edge. (A) The weighted adjacency matrix for the triangle graph. Rows and columns of the adjacency matrix are indexed by nodes of the triangle graph (i.e. by triangles in the information graph). The rows and columns of the matrix are sorted according to community order. Note the distinct block structure of the matrix indicating that the underlying graph is highly modular. (B) The same matrix, but unweighted so that the matrix contains only 0's and 1's (blue and red cells in the matrix, respectively) indicating that the nodes are either connected or disconnected. This aids in the visualization of the community structure of the graph (block structure of the matrix), although community detection was performed on the weighted triangle graph. (TIF) [file pcbi.1004005.s002.tif]

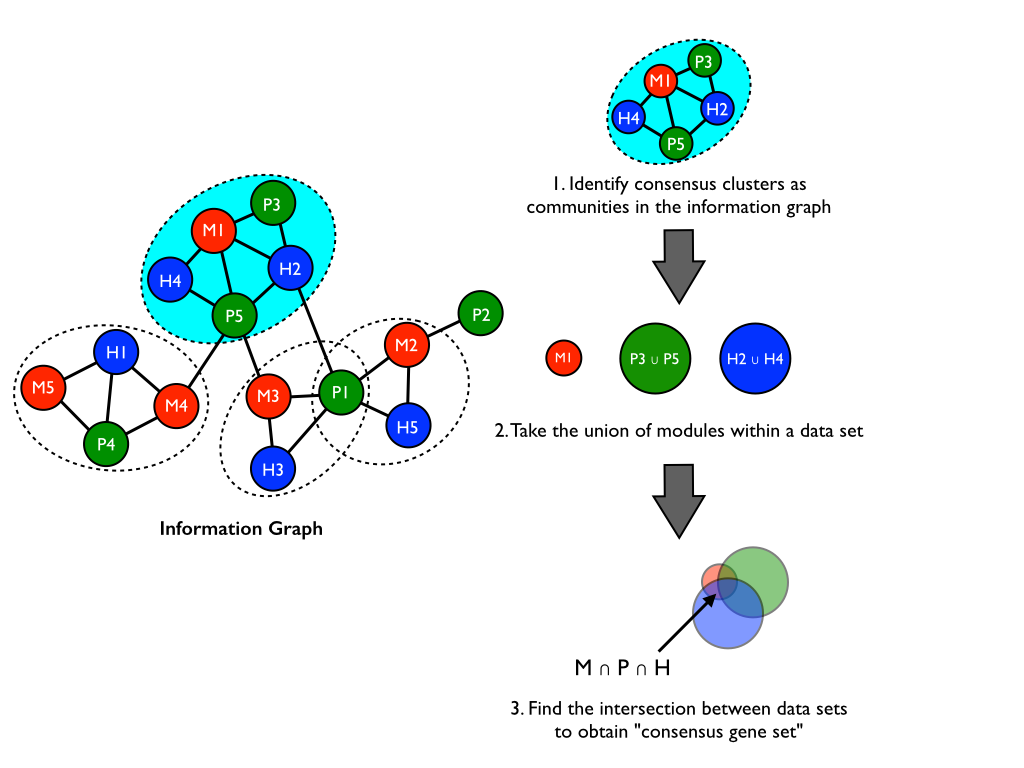

Supplement: S3 Fig — Schematic for building consensus gene sets. To each community (1) in the information graph we associate a consensus gene set by (2) computing the union of modules within a data set and then (3) computing the intersection across data sets. (TIF) [file pcbi.1004005.s003.tif]

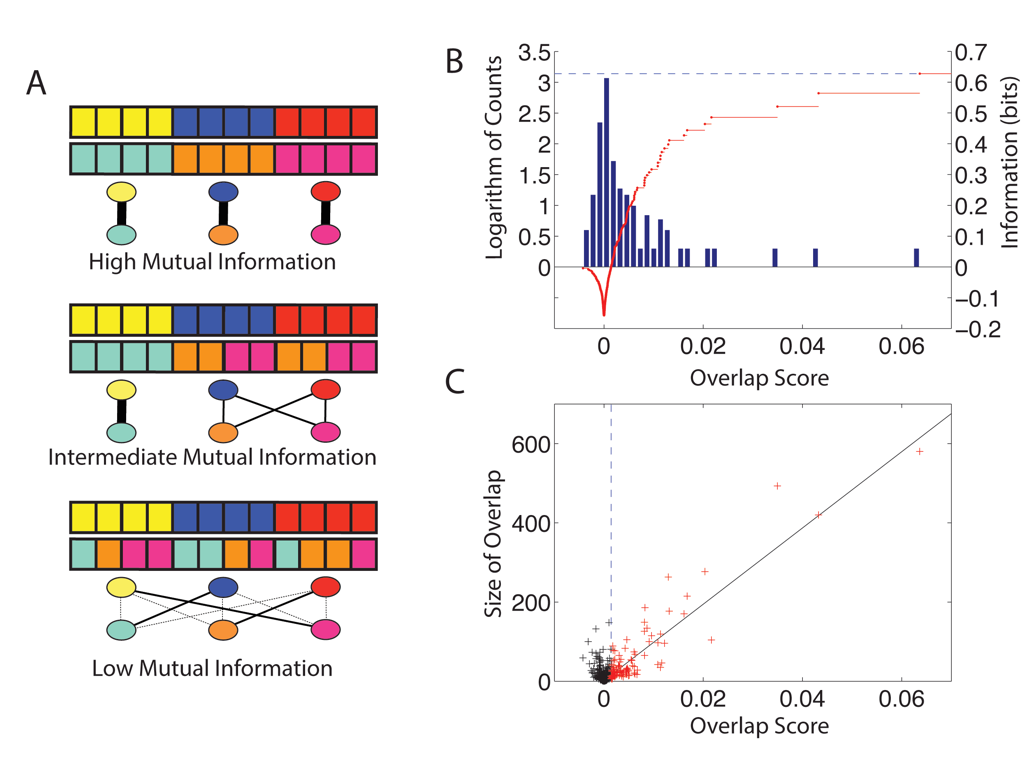

Supplement: S4 Fig — Construction of the information graph. (A) Three pairs of partitions of a 12-element set and their associated bipartite information graphs. Edge width denotes the size of the W-score for a pair of modules. Dotted edges represent negative W-scores. The highest possible mutual information occurs when modules are perfectly conserved. The information graph is disconnected with edges denoting the mapping between conserved modules. In the intermediate case, modules break into pieces that are reassorted among each other. The information graph here has strong community structure, but is not completely disconnected. The low mutual information case occurs when the partitions labels are random with respect to each other. In this case, all edges are small and are partially cancelled by the negative edges also present in the graph. (B,C) W-scores are calculated for each pair of modules; in this case one from Milano and one from Pendergrass. (B) Most W-scores are small in absolute value (blue histogram; logarithm of density), while their distribution has a right tail of significantly large scores. We can threshold the small and negative W-scores by keeping only those scores that contribute positively to the total mutual information (red curve; x-intercept). The sum of all W-scores is the total mutual information between the Milano and Pendergrass genomic partitions (dashed blue horizontal line). (C) The W-scores are positively correlated with the size of the overlap between gene clusters, but the relationship is not perfect. The W-score threshold is shown by a dotted blue vertical line and the overlaps that exceed the threshold are plotted in red. In particular, note that there are relatively large overlaps that fail to meet the threshold. Likewise, there are relatively small overlaps that have high W-scores. (TIF) [file pcbi.1004005.s004.tif]

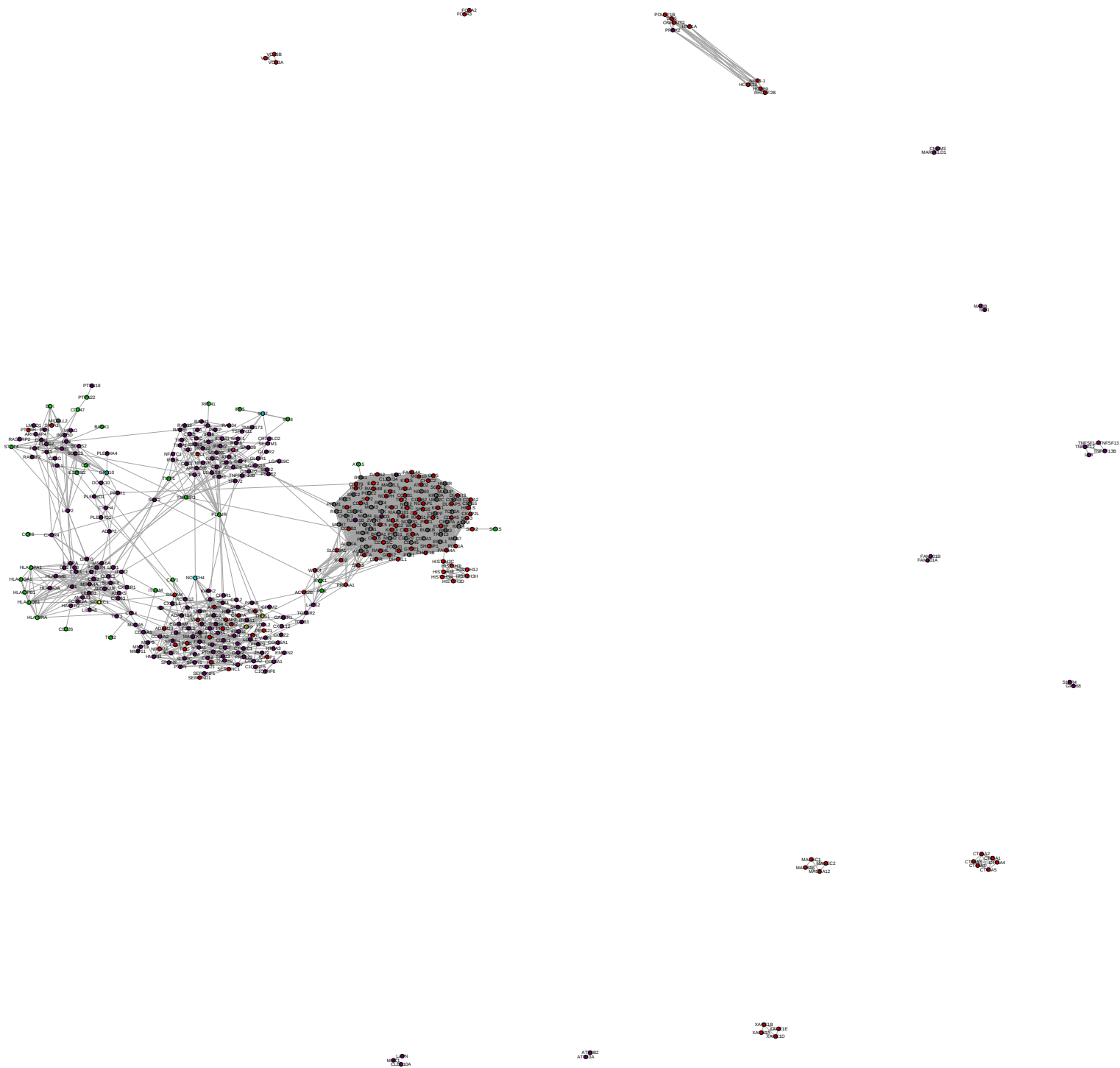

Supplement: S9 Data file — Molecular network plotted in PDF (text searchable for genes). (PDF) [file pcbi.1004005.s014.pdf]
