# Supplementary material for: Systems Level Analysis of Systemic Sclerosis Shows a Network of Immune and Profibrotic Pathways Connected with Genetic Polymorphisms
Source: PLoS Comput Biol. 2015 Jan 8;11(1):e1004005. doi: 10.1371/journal.pcbi.1004005 (PMC4288710; doi:10.1371/journal.pcbi.1004005)
Supplement: S1 Text — Additional mathematical details about the MICC method and glossary of keywords used in main text. (PDF) [file pcbi.1004005.s005.pdf]

Supplemental Methods

Mutual Information of Partitions

Mutual information of partitions (MIP) measures the similarity of two partitions of the same set, e.g. two independent clusterings of the genome into gene expression modules. MIP is maximized when two partitions are equal to each other and minimized when they are statistically independent of one another (Figure S2A). For more information about mutual information and its properties consult the excellent and comprehensive book [1] and for a comparison of mutual information based similarity to alternative similarity measures on the space of partitions of a finite set see [2].

Given two partitions P_1 and P_2 of a set S, mutual information quantifies the correlation of their cluster labels. Intuitively, MIP measures the sizes of the overlaps between clusters from P_1 and P_2 relative to the expected overlap size if the cluster labels were independent of each other. Mathematically MIP is defined as

$$MI(P_1,P_2) = \sum_{C_i \in P_1} \sum_{C_j \in P_2} \frac{|C_i \cap C_j|}{N} \log \frac{|C_i \cap C_j| N}{|C_i||C_j|}$$

Inspection of the summands in the above formula shows that the logarithmic factors are large and positive when the probability of a randomly drawn element of S being co-labeled by i and j is higher than expected under the assumption that the labels are independent. Likewise, the logarithmic factors are zero when the probability of co-labeling i and j is exactly the same as random and negative when the labels i and j "avoid" each other. The other factor in the summands is the joint probability of being co-labeled by i and j. If this factor is large, then the overlap of the ith and j^{th} clusters is large. Taken together these two factors give high weight to cluster overlaps that are large relative to the expectation of independent labels as well as absolute size relative to S. Thus, mutual information is well suited for use as a similarity measure between partitions because it incorporates both *relative* and *absolute* measures of the size of the overlap between two clusters.

Mutual information of partitions is distinguished for consensus clustering because of the mutual exclusivity of gene annotations to modules. A gene is always annotated to exactly one WGCNA module. At first glance, MIP is a sum of pairwise mutual information scores between two binary random variables: presence/absence of a gene in clusters C_i and C_j. The fact that our clusters form a partition forces dependency between the pairwise scores. The scores are not independent; moving a gene from one module to another must change *many* overlap scores. This means that p-values constructed using, say, hypergeometric tests for each pair of modules would yield correlated p-values and require a complicated multiple testing correction. The MIP framework allows us to sidestep that elegantly using a natural thresholding method (see below).

Construction of the Information Graph

The summands of Equation 1 is interpreted as a significance measure for the overlap of two clusters. Let C_i be a cluster in P_1 and C_j be a cluster in P_2. We define the "information weight" W_{ij} as

$$W_{ij} = \frac{|C_i \cap C_j|}{N} \log \frac{|C_i \cap C_j| N}{|C_i||C_j|}$$

where N = |S|, the total number of genes in the genome. W_{ij} is simply a single summand of Equation 1. We interpret W_{ij}'s as edge weights in a bipartite graph whose nodes are the clusters in P_1 and P_2. (The graph is bipartite because we assume clusters do not overlap within a partition and we use the convention that $0 \log_2(0) = 0$. Thus, there are no nonzero edges between clusters from the same partition.) For more than two partitions, we compute all pairwise W-scores and construct a k-partite graph in the same way we constructed the bipartite graph.

The W-scores can be negative and most are very small, indicating insignificant overlap between clusters. The scores below a threshold were set to zero. The W-scores were thresholded by computing the cumulative sum of the W-scores and keeping only the scores above the point where the sum becomes positive (Figure S2B red curve). For the current study, the information graph is the 3-partite graph obtained by computing all W-scores as above and setting all those scores that fell below the threshold to zero (Figure S2C).

We also compare the W-scores to the raw overlap sizes (i.e. the cardinality of the intersection between modules) in Figure S2C. The W-scores are correlated to the overlap sizes, but there is significant spread, particularly at the low end, indicating that the two measures disagree about which pairs deserve "small" scores. Because the W-score factors in the relative sizes of the two clusters to begin with, it gives low weight to a large overlap derived from a pair of huge clusters. Likewise, a small but perfectly conserved cluster gets a high W-score, but a low overlap score. Because of the scale-free nature of biological networks, the WGCNA modules vary in size over two orders of magnitude with some clusters containing a few thousand genes while others only tens of genes [3]. The W-scores are automatically regularized against this cluster size variation.

Triangle Percolation and Consensus Clusters

Community detection in the information graph was performed by a variant of clique percolation [4]. A minimal clique, i.e. a fully connected subset of nodes, in a tripartite graph is a triangle. Clique percolation is a community detection technique that is based on the observation that cliques within a community will with high likelihood be "adjacent" to other cliques. For example, edge percolation (2-clique percolation) finds communities in a network by finding sets of edges that share many vertices. Likewise, triangle percolation (3-clique percolation) finds communities by looking for sets of triangles with many edges in common. Triangles have the additional interpretation of a

module that is approximately shared across all datasets. Thus, triangle percolation is a natural community detection algorithm.

To perform triangle percolation, we enumerated all triangles in the information graph. This can be accomplished by greedy search. With the full set of triangles in the information graph, we formed the “triangle graph" which is simply a graph whose nodes represent triangles in the information graph and whose edges indicate that the corresponding triangles share an edge in the information graph. We weighted the edges of the triangle graph by the weight of the shared edge of the triangles. This allows high weight edges to strongly influence community formation. We found communities in the triangle graph by modularity maximization [5]. The communities of triangles correspond to sets of modules from each dataset that are approximately preserved across the three datasets.

To derive a gene set associated to the triangle communities, we took all modules within that community, computed their unions within their dataset, and then computed their intersection across datasets. We call this final gene set a *consensus cluster* and its elements *consensus genes*. Note that, by the distributivity of intersection over union, this is equivalent to taking the three-way intersections between all modules in the community and then the union of those intersections. A more conservative alternative would be to take only the union of those intersections that derive from actual triangles in the community and not all possible triangles that can be made from the modules in the community. We opted against this because the presence of a gene in a module means that *there exists a context* in which that gene is coexpressed with the others in the module, thus the more liberal option cannot introduce genes that are wildly irrelevant. Moreover, the choice we have made here is extensible to any definition of community in the information graph, even if that (in general) k-partite graph has few or no k-cliques. This may be important in future studies using MICC involving many data sets.

Hubs and consensus genes

A module from WGCNA can be summarized by its module eigengene, which is the first principal component of the gene-normalized gene expression matrix for the genes in the module. The module eigengene is a theoretical construct that represents the hub gene of the module [6]. Genes that are highly correlated to the eigengene are thus more central to the module. The consensus genes from MICC are substantially more correlated to their eigengenes than randomly chosen genes (Figure S1). Thus the consensus clusters are enriched for hubs in their corresponding gene-gene coexpression networks. This is a useful proof-of-principle that MICC is identifying relevant structure in the data.

Glossary of terms used in this paper

The MICC analysis pipeline is motivated by the need to integrate several bioinformatics analyses across multiple datasets. Here we collect a set of terms that: 1) are used in a technical sense in MICC, 2) are standard in the bioinformatics/machine learning literature, and 3) are outside the scope of the main text.

Network/Graph

Networks are *any* collections of objects (called **nodes**) that have relationships with each other (called **links**). The nature of the nodes and links in a network determines the character of the network and what information it encodes. A standard, alternative term for a network is a graph. The terms network and graph are used interchangeably, but typically we use network when the nodes represent physically real things (like genes or proteins), whereas we use graph when the nodes are an abstraction of some sort.

In the MICC method, there are 3 distinct types of networks that are used. They are given here in order of abstraction:

1) Gene-gene coexpression networks derived from a single gene expression data cohort: nodes are genes, links are correlations between expression patterns
2) The information graph: nodes are modules derived from the gene-gene coexpression networks, links are similarity scores between modules derived from *different* datasets. A link indicates that the two modules have a larger than expected overlap if the two gene-gene expression networks were completely dissimilar. Note that links only connect modules from different datasets.
3) The triangle graph: nodes are triangles in the information graph, links are shared edges between triangles. A triangle in the information graph indicates that there are modules in each of the three datasets that are significantly similar to each other. In other words, a triangle in the information graph indicates that there is a core set of genes whose gene expression is preserved across all datasets. The triangle graph takes this one step further by considering the *relationships* between triangles. The triangle graph encodes not just perfect conservation of modules, but approximate conservation.

There is one further network that is used in the main manuscript, the IMP Bayesian functional network. The nodes of the IMP network are genes and the links represent high probability functional interactions between those genes (e.g. the genes form a complex under some circumstance). The IMP network is a network learned from all publicly available gene expression data. It is not the same as a gene-gene coexpression network because its links represent probabilities, not correlations.

Node

Nodes are the basic unit of a network. They are the objects whose relationships are encoded in the network. For example, nodes could be genes and the network encodes some notion of relationship between genes.

Link/Edge
Links are the unit of *relationship* between nodes in a network. A standard, alternative term for a link is an edge. Links denote that a pair of nodes is related. Links in our case are *weighted* meaning that they denote the strength of a relationship between nodes. For example, links in a gene-gene network could represent the correlation of those genes in a particular experiment. The weight in this case is the strength of the correlation.

Cluster/Clustering
A cluster is any grouping of objects by a notion of similarity between objects. There are two notions of cluster used in MICC: gene expression clusters and consensus clusters. The former are sets of genes that are similar in the sense that they are coexpressed *within* a single microarray dataset. The latter are sets of the gene expression modules that are similar in the sense that they a broadly conserved *across* datasets.

Clustering is any algorithmic procedure that identifies groups of similar objects.

Module
A module is an alternative term for a cluster of genes that are grouped together by coexpression. Module is the standard term applied the output of Weighted Gene Coexpression Network Analysis (WGCNA).

Consensus cluster
A consensus cluster is set of genes whose coexpression is preserved across multiple datasets. MICC is a procedure for *identifying* these sets of genes.

Partition
A partition is a grouping of elements of a set into distinct groups. For example, WGCNA forms a partition of the genome by clustering genes into distinct, non-overlapping modules. The whole collection of modules from WGCNA comprises the partition.

Information graph
The information graph is network whose nodes are modules from distinct datasets and links represent a significantly large overlap between those modules.

Communities
A community is a subgroup of nodes in a network that are more densely interconnected to each other than they are to the rest of the network. Two “community detection” procedures are used in MICC: 1) WGCNA is a state-of-the-art algorithm for detecting communities specifically in gene coexpression networks; the communities in this case are called modules. 2) Clique percolation is a generic community detection procedure applicable for an arbitrary network. MICC uses clique percolation in the triangle graph to find sets of triangles (in the information graph) that share many edges. The communities in this case can be used to derive a consensus cluster gene set.

WGCNA

Weighted gene coexpression network analysis (WGCNA) is a clustering procedure takes gene expression data from a single cohort and finds groups of genes that are highly correlated to each other and weakly correlated outside of their group. WGCNA is built upon the notion of a gene coexpression network (see above) and extracts a small set of signals that account for a large fraction of the gene expression variance.

Principal components/Module eigengenes

Principal component analysis (PCA) is a procedure for simplifying high-dimensional data and summarizing it with fewer dimensions (e.g. to plot in two dimensions). In this paper, we used PCA to extract the first principal components of gene expression modules. The output of WGCNA is a set of gene clusters (modules). The gene expression of any gene within a module is very similar to that of any other gene in the same module. Thus, we can capture the major signal in the gene expression of the whole module by simply considering the *first* principal component of the module's full gene expression profile. This principal component is called the "module eigengene". The term eigengene is related to the fact that PCA is performed using eigenvalues/eigenvectors from linear algebra.

Hub

A hub is a node in a network that is extremely densely connected to the rest of the network (or a part of the network). Biological networks have a well-studied property (called "scale-free") where most nodes (genes, proteins, etc.) are weakly connected to the rest of the network, but a small fraction of the nodes are extremely highly connected. These highly connected nodes are called hubs. It has been shown that hubs in biological networks are key molecules involved in their various biological functions, without which the system is severely impaired.

There are two types of hubs we consider in this paper. First, hubs in gene-gene coexpression networks are genes whose expression is highly correlated to the expression of many other genes. The module eigengenes from WGCNA represent theoretical genes that are the hubs of their respective module. The module eigengene, if it were a real gene, would be the most connected gene in the module. Genes that are very similar to an eigengene are therefore very highly correlated to the other genes within their module.

The other type of hub we consider are those in the IMP Bayesian functional network. Again, in the IMP network nodes are genes, but the links are (predicted) interactions between the genes. In this case, then, a hub is a gene that has a very high number of interactions with other genes.

References

1. Cover TM, Thomas JA (2012) Elements of information theory: Wiley-interscience.
2. Meila M (2003) Comparing clusterings by the variation of information. Learning theory and kernel machines: Springer. pp. 173-187.

3. Langfelder P, Horvath S (2008) WGCNA: an R package for weighted correlation network analysis. BMC Bioinformatics 9: 559.
4. Palla G, Derenyi I, Farkas I, Vicsek T (2005) Uncovering the overlapping community structure of complex networks in nature and society. Nature 435: 814-818.
5. Newman MEJ (2006) Modularity and community structure in networks. Proceedings of the National Academy of Sciences 103: 8577-8582.
6. Horvath S, Dong J (2008) Geometric interpretation of gene coexpression network analysis. PLoS Comput Biol 4: e1000117.
